# Endothelial-mesenchymal transition harnesses HSP90α-secreting M2-macrophages to exacerbate pancreatic ductal adenocarcinoma

**DOI:** 10.1186/s13045-019-0826-2

**Published:** 2019-12-17

**Authors:** Chi-Shuan Fan, Li-Li Chen, Tsu-An Hsu, Chia-Chi Chen, Kee Voon Chua, Chung-Pin Li, Tze-Sing Huang

**Affiliations:** 1 National Institute of Cancer Research, National Health Research Institutes, No. 35, Keyan Road, Zhunan Township, 350 Miaoli, Taiwan; 2 Institute of Biotechnology and Pharmaceutical Research, National Health Research Institutes, No. 35, Keyan Road, Zhunan Township, 350 Miaoli, Taiwan; 3 Division of Gastroenterology and Hepatology, Department of Medicine, Taipei Veterans General Hospital, Taipei, Taiwan; 4 School of Medicine, National Yang-Ming University, Taipei, Taiwan; 5 Department of Biochemistry, School of Medicine, Kaohsiung Medical University, Kaohsiung, Taiwan; 6 Program in Tissue Engineering and Regenerative Medicine, Biotechnology Center, National Chung Hsing University, Taichung, Taiwan

**Keywords:** EndoMT, Cancer-associated fibroblast, M2-type macrophage, eHSP90α

## Abstract

**Background:**

Endothelial-to-mesenchymal transition (EndoMT) can provide a source of cancer-associated fibroblasts which contribute to desmoplasia of many malignancies including pancreatic ductal adenocarcinoma (PDAC). We investigated the clinical relevance of EndoMT in PDAC, and explored its underlying mechanism and therapeutic implication.

**Methods:**

Expression levels of 29 long non-coding RNAs were analyzed from the cells undergoing EndoMT, and an EndoMT index was proposed to survey its clinical associations in the PDAC patients of The Cancer Genome Atlas database. The observed clinical correlation was further confirmed by a mouse model inoculated with EndoMT cells-involved PDAC cell grafts. In vitro co-culture with EndoMT cells or treatment with the conditioned medium were performed to explore the underlying mechanism. Because secreted HSP90α was involved, anti-HSP90α antibody was evaluated for its inhibitory efficacy against the EndoMT-involved PDAC tumor.

**Results:**

A combination of low expressions of LOC340340, LOC101927256, and MNX1-AS1 was used as an EndoMT index. The clinical PDAC tissues with positive EndoMT index were significantly correlated with T4-staging and showed positive for M2-macrophage index. Our mouse model and in vitro cell-culture experiments revealed that HSP90α secreted by EndoMT cells could induce macrophage M2-polarization and more HSP90α secretion to promote PDAC tumor growth. Furthermore, anti-HSP90α antibody showed a potent therapeutic efficacy against the EndoMT and M2-macrophages-involved PDAC tumor growth.

**Conclusions:**

EndoMT cells can secrete HSP90α to harness HSP90α-overproducing M2-type macrophages to promote PDAC tumor growth, and such effect can be targeted and abolished by anti-HSP90α antibody.

## Background

Pancreatic ductal adenocarcinoma (PDAC) is the most common pancreatic cancer, exhibiting conspicuous desmoplasia and deadly prognosis [1]. The desmoplastic stroma of PDAC is composed of large amounts of extracellular matrix as well as great numbers of α-smooth muscle actin (α-SMA)-expressing myofibroblastic cells. Such myofibroblastic cells, also called activated fibroblasts or cancer-associated fibroblasts (CAFs), contribute to tumor growth, immunosuppression, and malignant progression [2–5]. They constitute the majority of tumor stromal cells and can be derived from diverse resources such as tissue-resident fibroblasts, stellate cells, mesenchymal stem/progenitor cells, and infiltrating fibrocytes [6]. Additionally, 30~40% of CAFs can arise from the endothelial-to-mesenchymal transition (EndoMT) of endothelial cells [7], exhibiting a remarkable cancer-associated cell plasticity. EndoMT is first observed with heart development [8–10], and is also involved in transforming growth factor (TGF)-β-associated fibrotic diseases [11]. Despite the fact that EndoMT can be detected in cancers and is thought as a source of CAFs, the knowledge about the relevance of EndoMT to other clinical characteristics and the underlying mechanism(s) is still lacking. In our previous study, EndoMT cells (exhibiting α-SMA^+^ and CD31^+^) were detected nearby osteopontin (OPN)-expressing macrophages in colorectal cancer (CRC) tissue specimens [12]. OPN induced the EndoMT of endothelial cells, and the resultant EndoMT cells exhibited a potent tumor-promoting effect by secreting HSP90α to foster the stemness of CRC cells [12]. HSP90α is a well-known cellular chaperone aiding the folding, maturation, and trafficking of many client proteins including cancer-related Bcr-Abl, ErbB2/Neu, Akt, HIF-1α, mutated p53, and Raf-1 [13]. It can also be expressed and secreted from the keratinocytes and fibroblasts in wounded tissues, as well as from cancer cells [14–16]. Clinically, elevation of serum/plasma HSP90α levels has been detected from several malignancies including CRC and PDAC [15–18]. Elevated levels of such extracellular HSP90α (eHSP90α) can also be detected from pancreatitis patients and PDAC-developing transgenic mice driven by mutant K-Ras [18]. eHSP90α could be produced from myeloid-derived macrophages and the stimulated pancreatic ductal epithelial cells to promote the macrophage-associated PDAC development [18]. Macrophages are one of the most abundant myeloid-derived cells infiltrating the tumor microenvironment. Earlier studies have demonstrated that inflammatory macrophages have tumoricidal activity, but macrophages polarize to M2-type and exhibit distinct tumor-promoting activities after interacting with tumor cells and other components within the tumor microenvironment [19, 20]. Higher level of M2-macrophages has been clinically correlated with PDAC malignancy [18, 21], while a significant correlation between the levels of M2-macrophage and CAF was also revealed in CRC tissues [22]. However, the relations among EndoMT, M2-macrophages, eHSP90α level, and PDAC malignancy remain poorly disclosed.

To investigate EndoMT-associated PDAC microenvironment and clinical significance, we first identified an expression profile of 3 long non-coding RNAs (lncRNAs) as EndoMT index to characterize the clinical PDAC specimens in TCGA dataset. The PDAC tissues with positive EndoMT index were significantly correlated with T4-staging and showed positive for M2-macrophage index. Furthermore, our mouse model and in vitro co-culture experiments revealed that HSP90α secreted by EndoMT cells was able to induce macrophage M2-polarization and more HSP90α secretion to promote PDAC tumor growth. Anti-HSP90α antibody exhibited a potent therapeutic efficacy against the EndoMT-promoted and M2-macrophage-involved PDAC tumor.

## Methods

### Clinical tissue specimens

Besides using the data of 177 PDAC tissues enrolled in TCGA dataset, we studied the PDAC tissues taken from 12 patients who received surgery at Taipei Veterans General Hospital (Taipei, Taiwan), and written informed consent was obtained from each patient according to the medical ethics protocol approved by the Human Clinical Trial Committee of Taipei Veterans General Hospital.

### Cell cultures

Human umbilical vein endothelial cells (HUVECs) were isolated from umbilical cords of normal deliveries upon approval by the Human Clinical Trial Committee of Taipei Veterans General Hospital, and cultured in a 37 °C and 5% CO_2_ humidified incubator with M199 medium plus 20% of fetal bovine serum (FBS), 30 μg/ml of endothelial cell growth supplement (EMD Millipore, Billerica, MA), 100 units/ml of penicillin, and 100 μg/ml of streptomycin [23]. For EndoMT induction, HUVECs were pre-incubated 16 h with 2% FBS-containing M199 medium and then added with 0.3 μg/ml of OPN for further 24 h. Human immortalized endothelial cell line EC-RF24 [24] and mouse immortalized endothelial cell line 3B-11 (ATCC CRL-2160; American Type Culture Collection, Manassas, VA) were grown in RPMI 1640 medium supplemented with 10% FBS and a mixture of 100 units/ml of penicillin, 100 μg/ml of streptomycin, and 2 mM of L-glutamine (1× PSG). For EndoMT induction, these cells were treated same as above except for 1% FBS-containing RPMI 1640 medium was used instead. Human monocytic leukemia THP-1 cells were cultivated in RPMI 1640 medium supplemented with 10% FBS and 1× PSG. To induce differentiation and polarization, THP-1 cells were pre-incubated 6 h with the culture medium containing 100 ng/ml of 12-*O*-tetradecanoyl-13-phorbol acetate (TPA) and then added with 100 ng/ml of lipopolysaccharide (LPS) plus 20 ng/ml of interferon-γ (IFN-γ) as M1-inducer or 20 ng/ml of interleukin (IL)-4 plus 20 ng/ml of IL-13 as M2-inducer for another 24-h incubation. Mouse immortalized macrophage line RAW264.7 were cultured with Dulbecco’s modified Eagle’s medium (DMEM) plus 10% FBS and 1× PSG, and treated same as above for M1/M2 polarization. For mouse bone marrow-derived macrophage (BMDM) preparation, bone marrow cells were isolated from C57BL/6 mice and incubated with DMEM supplemented with 10% FBS, 20% L929-conditioned medium, and 1× PSG for 7 days. Adherent BMDMs were maintained in DMEM plus 10% FBS and 1× PSG. Human PDAC cell lines PANC-1 and MIA PaCa-2 were cultivated with DMEM plus 10% FBS and 1× PSG. Human PDAC cell line AsPC-1 and mouse PDAC cell line Panc 02 were cultivated with RPMI 1640 medium plus 10% FBS and 1× PSG.

### Preparation of conditioned media (CM)

To prepare the CM of endothelial and EndoMT-derived cells, HUVECs, EC-RF24, and 3B-11 cells (2 × 10^6^ cells/10-cm dish) were treated 24 h with control PBS or OPN in their respective low-serum media as described above. After washing twice with PBS, control and OPN-treated cells were incubated with 5 ml of fresh low-serum media for another 24 h. Dishes of the respective low-serum media without cells were prepared simultaneously as control media. The media were collected, filtrated with 0.45-μm filters, and designated as “Ctrl,” “Endo CM,” and “EndoMT CM,” respectively. To prepare the CM of rHSP90α or EndoMT-affected human macrophages, THP-1 cells were treated with 100 ng/ml of TPA for 24 h and then adherent cells were collected and seeded at a density of 2 × 10^6^ cells per 10-cm dish. Furthermore, the adherent macrophages were pre-incubated with 1% FBS-containing RPMI 1640 medium for 16 h. The media were then added with PBS or 15 μg/ml of rHSP90α (Enzo Life Sciences Inc., Farmingdale, NY) or replaced with “Ctrl,” “Endo CM,” or “EndoMT CM” for further 24 h. After washing twice with PBS, treated macrophages were incubated with 5 ml of fresh 1% FBS-containing media for 24 h. The media were collected and filtrated with 0.45-μm filters. To prepare the CM of EndoMT-affected mouse macrophages, RAW264.7 cells were pre-incubated with 1% FBS-containing DMEM for 16 h and then incubated with “Ctrl,” “Endo CM,” and “EndoMT CM,” respectively, for another 24 h. After PBS washes, treated RAW264.7 cells were incubated with 5 ml of fresh 1% FBS-containing DMEM for 24 h. The media were collected and subjected to filtration through 0.45-μm filters.

### Mouse model

All mouse experiments were performed with C57BL/6 mice (6~7 week-old), which were approved by the Institutional Animal Care and Use Committee of National Health Research Institutes. For tumor transplantation, 1 × 10^6^ Panc 02 cells were mixed with Matrigel plus 2.5 × 10^5^ 3B-11 cells pretreated 24 h with PBS or 0.3 μg/ml of OPN before subcutaneously injected into mice on day 0. Sizes of developing tumors were superficially measured with a Vernier caliper every 3 days, and tumor volumes were calculated with the formula 1/2 × length × width^2^. Mice were sacrificed on day 30 and tumors were removed and weighed. For evaluation of the tumor-suppressive efficacy of anti-HSP90α antibody, the mice inoculated with Panc 02 cells plus OPN-treated 3B-11 cells were further intravenously injected with control IgG or anti-HSP90α antibody (5 μg per g of body weight for each dosage) on day 4. The injections were performed 8 times at 3-day intervals. The anti-HSP90α monoclonal antibody was prepared by LTK Biotechnologies (Taoyuan, Taiwan).

### Immunohistofluorescence (IHF)

Paraffin-embedded tissue sections with a 4-μm thickness were deparaffinized by xylene and rehydrated by graded ethanol dilutions. For antigen retrieval, these tissue sections were heated for 15 min in 10 mM citrate buffer, pH 6.0, under high pressure and then blocked in 3% BSA-containing PBS for 30 min at room temperature. For human tissue staining, the tissue sections were incubated overnight at 4 °C with primary antibodies (Additional file 1: Table S1). After washing with PBS plus 0.1% Tween-20, the corresponding secondary antibodies were applied. After incubation at room temperature for 1 h, nuclei were stained with 4′,6 ′-diamidino-2-phenylindole (DAPI). For mouse tissue staining, one set of tissue sections were incubated with mouse anti-CK18 antibody, rabbit anti-α-SMA antibody, and goat anti-CD31 antibody at room temperature for 60 min. Another set of tissue sections were incubated with rat anti-F4/80 antibody at room temperature for 60 min and then with rabbit anti-iNOS antibody plus mouse anti-Arg1 antibody or with rabbit anti-MHC II antibody plus mouse anti-CD163 antibody at room temperature for another 60 min. After primary antibody incubation, the tissue sections were washed twice with PBS plus 0.1% Tween-20 and incubated with respective fluorescence-labeled secondary antibodies (Additional file 1: Table S1) for 30 min at room temperature. Nuclei were then stained with DAPI and finally, results were observed, analyzed, and photographed under Leica TCS SP5 II confocal microscope and LASAF software (Leica, Wetzlar, Germany).

### Immunohistochemistry (IHC)

Mouse tissue sections with a 4-μm thickness were deparaffinized by xylene, rehydrated through a series of ethanol dilutions, heated in 10 mM citrate buffer, pH 6.0, and inactivated endogenous peroxidase activity by 0.3% H_2_O_2_. These tissue sections were then blocked with 3% BSA and incubated 60 min at room temperature with primary antibodies (Additional file 1: Table S1). After washing with PBS plus 0.1% Tween-20, the secondary antibodies were applied at room temperature for 30 min. Finally, these tissue sections were subjected to detection using the DAKO REAL EnVision Detection System (Produktionsvej 42, DK-2600 Glostrup, Denmark) and counterstained with hematoxylin.

### RNA extraction and RT-PCR

Cellular total RNA was extracted using TRIzol reagent (Thermo Fisher Scientific, Waltham, MA). One microgram of RNA was converted to cDNA by Tetro Reverse Transcriptase (Bioline Reagents Ltd., London, UK). The cDNA products were used as the templates for PCR analyses. The primers and reaction conditions were summarized in Additional file 1: Table S2. Real-time quantitative PCR (qPCR) was performed with QuantiNova SYBR Green RT-PCR Kit (Qiagen, Hilden, Germany) in StepOnePlus^TM^ Real-Time PCR System (Thermo Fisher Scientific).

### Transendothelial migration assay

In mouse cell model, endothelial cell layers were prepared by seeding 3B-11 cells (3 × 10^5^) into each Transwell^TM^ insert (pore size, 8 μm; BD Biosciences, San Jose, CA) precoated with 50 μl of 1/3 diluted Matrigel^TM^ (BD Biosciences). RAW264.7 cells with or without induction of M1 or M2-polarization (1 × 10^5^ cells) were labeled with fluorescence by incubating 20 min with 7.5 M of carboxyfluorescein diacetate (CFDA, Invitrogen, Carlsbad, CA). Furthermore, CFDA-labeled macrophages were seeded onto the endothelial cell layer in each Transwell^TM^ insert. On the other hand, Panc 02 cells (8 × 10^5^) alone or together with 3B-11 or OPN-treated 3B-11 cells (2 × 10^5^) were seeded per well of 24-well plates. Each Transwell^TM^ insert was then placed into the well of a 24-well plate, and the entire set-up was incubated in a 37 °C and 5% CO_2_ humidified incubator for 16 h. The remaining macrophages together with the Matrigel^TM^ in the Transwell^TM^ insert were gently swabbed out using cotton swabs. The macrophages migrating onto the lower side of the Transwell^TM^ insert were observed and counted under the Axiovert S100/AxioCam HR microscope system (Carl Zeiss, Oberkochen, Germany). In human cell model, experimental conditions were same as those in mouse cell model except for 3B-11, RAW264.7, and Panc 02 cells were replaced by HUVEC, THP-1-derived, and PANC-1 cells, respectively.

### Enzyme-linked immunosorbent assay (ELISA)

The amounts of IL-1β, IL-10, and TGF-β1 in medium samples were measured according to the manufacturer’s protocol of ELISA kits (R&D Systems, Minneapolis, MN). Briefly, 100 μl of standards and samples were loaded per well of 96-well plates. After incubation with biotinylated antibodies, streptavidin-conjugated horseradish peroxidase (HRP) was added to each well and followed by HRP substrate solution. Similarly, quantitative determination of the secreted HSP90α levels of medium and serum samples was performed as described previously [17]. Finally, the enzyme reactions were stopped and the OD_450_ values were detected using Infinite M200 microplate reader (TECAN, Männedorf, Switzerland).

### Flow cytometry

THP-1 cells were treated with 100 ng/ml of TPA for 24 h, and adherent cells were collected and re-seeded at a density of 4 × 10^6^ cells per 10-cm dish. Furthermore, the adherent macrophages were pre-incubated with 1% FBS-containing RPMI 1640 medium for 16 h. The media were then added with 15 μg/ml of rHSP90α or replaced with “Ctrl,” “Endo CM,” or “EndoMT CM” for another 24 h. After washing with PBS, treated macrophages were trypsinized and collected, and 1 × 10^5^ cells were resuspended in 50 μl of 4 °C PBS plus 1% FBS added with primary antibodies (Additional file 1: Table S1) for a further 60-min incubation. Subsequently, cells were washed twice with PBS and then stained 40 min with the respective secondary antibodies. After washing, cells were immediately analyzed by FACSCalibur flow cytometer (BD Biosciences).

### Cell lysate preparation and immunoblot analysis

Cell lysates were prepared by briefly sonicating cells in lysis buffer [18] plus cocktails of protease inhibitors and phosphatase inhibitors (Sigma-Aldrich, St. Louis, MO). The protein concentrations of cell lysates were determined by BCA protein assay kit (Thermo Fisher Scientific), and immunoblot analyses were performed according to the procedure described previously [17]. The protein bands were detected by enhanced chemiluminescence (Luminata^TM^ Crescendo Western HRP Substrate, EMD Millipore). The antibodies used were listed in Additional file 1: Table S1.

### Proximity ligation assay (PLA)

Macrophages were seeded on glass coverslips at a density of 2 × 10^5^ cells per ϕ12-mm coverslip. After pre-incubating 16 h with 1% FBS-containing RPMI 1640 medium, the macrophages were added with PBS or 15 μg/ml of rHSP90α for a further 24-h incubation. The subsequent PLA was performed according to the manufacturer’s instructions of the Duolink in situ PLA kit (Sigma-Aldrich). The antibodies used for this study were listed in Additional file 1: Table S1. The final images were photographed and analyzed by TCS SP5 II confocal microscope and LASAF software (Leica).

### Chromatin immunoprecipitation (ChIP)

ChIP assay was performed based on the manufacturer’s instruction of EZ-ChIP kit (EMD Millipore). Briefly, PBS or rHSP90α-treated macrophages were treated with 1% formaldehyde for cross-linking and then subjected to cell lysis and DNA fragmentation. After preclearing with protein G-conjugated agarose, 10-μl aliquots of cell lysates were saved as “input” fractions, and the remaining lysates were added with control IgG or anti-STAT-3 antibody for immunoprecipitation. Furthermore, DNA was extracted from the immunoprecipitates for PCR analysis of the STAT-3 site-containing region of *HSP90α* gene promoter. The primers and condition were as follows: forward, 5′-GGT-GAA-ACC-CCG-ACT-CTA-CA-3′; reverse, 5′-GCC-TCA-GCT-TTC-CCA-GTA-GC-3′; 95 °C (30 sec), 64 °C (40 sec), and 72 °C (30 sec) for 38 cycles.

### Statistical analysis

Cell culture experiments were performed at least three times. Results of cell culture experiments and mouse model were analyzed by independent samples *t* test. The Pearson *χ*^2^ test was used to analyze the relationships of tumor EndoMT index with α-SMA^high^ and CD31^high^ status, patient’s AJCC T-staging, and tumor M2-macrophage index. The differences were considered significant if *P <* 0.05.

## Results

### EndoMT is preferably detected in T4-staging and M2-macrophage-infiltrating PDAC tissues

**Fig. 1:**
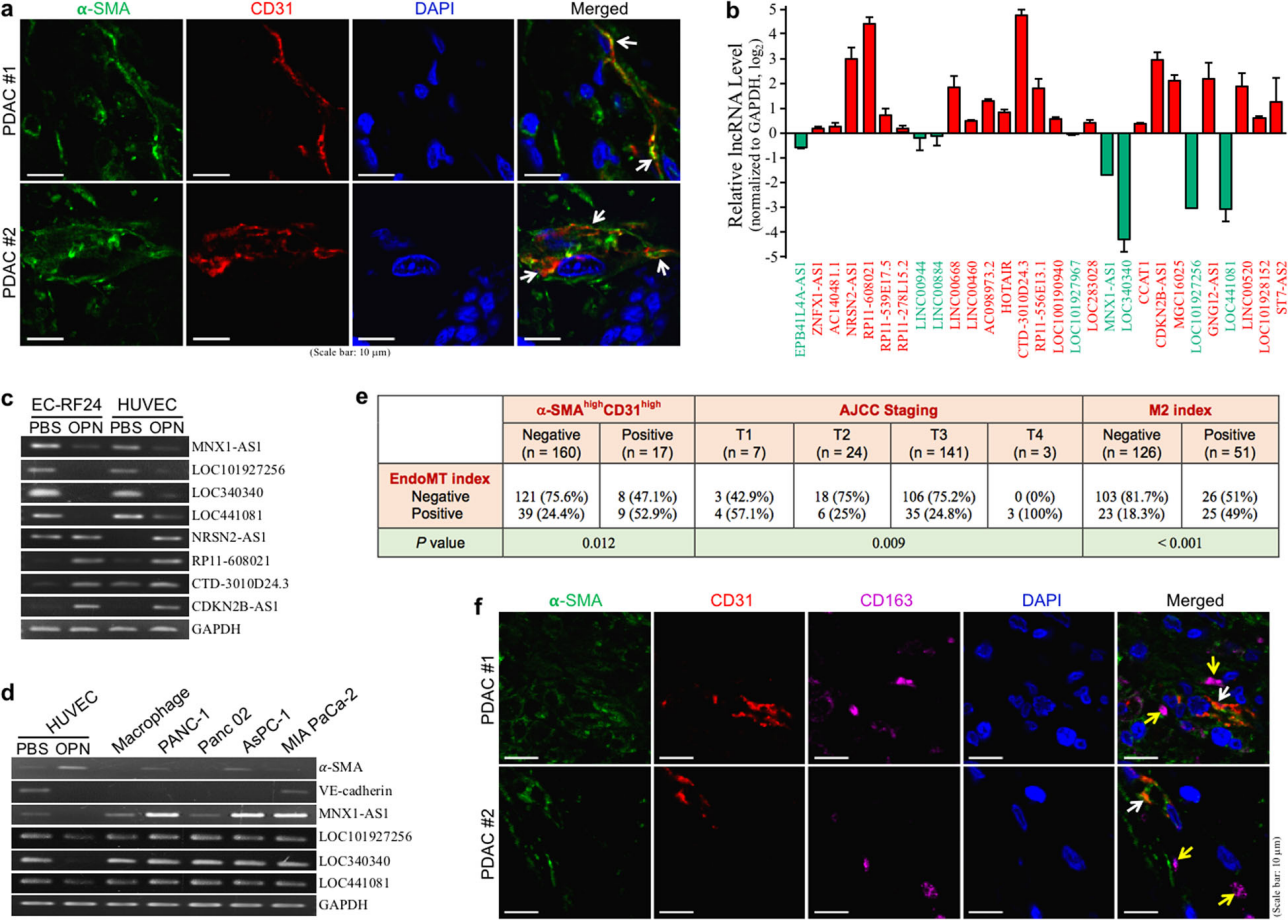
EndoMT occurs significantly with M2-macrophage infiltration in PDAC tissues. **a** IHF of α-SMA and CD31 from the tumor tissues of PDAC patients. Nuclei were stained with DAPI. EndoMT-derived cells exhibiting α-SMA^+^ and CD31^+^ are indicated by arrows. **b** Changes of the lncRNA levels in EndoMT cells derived from HUVECs. Among the 29 lncRNAs analyzed by qPCR, 21 of them were upregulated and only 8 were downregulated. **c** Downregulation of MNX1-AS1, LOC101927256, LOC340340, and LOC441081 and upregulation of RP11-608021, CTD-3010D24.3, and CDKN2B-AS1 in EndoMT cells derived from OPN-treated HUVECs and human immortalized endothelial cell line EC-RF24 cells. **d** Low expression of MNX1-AS1, LOC101927256, LOC340340, and LOC441081 in HUVEC-derived EndoMT cells compared with PDAC cells and macrophages. **e** Correlations of the EndoMT level of PDAC tissues with T4 staging and M2-macrophage infiltration. The data of 177 PDAC patients in TCGA database were analyzed to reveal the clinical relevance of the EndoMT occurrence. Using average expression values as cut-off points, we proposed a combination of low expressions (< averages) of LOC340340, LOC101927256, and MNX1-AS1 as the EndoMT index, and observed that PDAC tissues with positive EndoMT index were significantly correlated with T4 staging and positive for M2-macrophage index (CD163^high^ and CD204^high^). The data of AJCC staging were available from 175 patients. **f** IHF of α-SMA, CD31, and CD163 showing EndoMT-derived cells (indicated by white arrows) and neighboring M2-type macrophages (indicated by yellow arrows) in the tumor tissues of PDAC patients. Nuclei were stained with DAPI

EndoMT cells exhibiting α-SMA^+^ and CD31^+^ can be detected from cancer tissues of PDAC patients (Fig. 1a). To further decipher their clinical relevance, we intended to find a molecular EndoMT index that can be easily used to characterize clinical PDAC specimens. EndoMT which can be induced by treating endothelial cells with OPN as studied previously exhibits a lncRNA expression profile shown in Fig. 1b. Among these 29 lncRNAs, 21 of them were upregulated, whereas only 8 were downregulated. Nine upregulated lncRNAs including CTD-3010D24.3, RP11-608021, CDKN2B-AS1, and NRSN2-AS1 were increased by at least threefolds, while the top 4 downregulated lncRNAs LOC340340, LOC101927256, LOC441081, and MNX1-AS1 had more than threefold decreases. Among these changes, downregulation of LOC340340, LOC101927256, LOC441081, and MNX1-AS1 can be detected in EndoMT cells derived from both HUVECs and immortalized endothelial cell line EC-RF24 (Fig. 1c). The downregulation was observed only in EndoMT cells despite of high levels of expression in PDAC cells and macrophages (Fig. 1d). Therefore, a combination of low expressions of LOC340340, LOC101927256, and MNX1-AS1 was used as a potential EndoMT index to classify 177 PDAC patients in TCGA database. Positive EndoMT index was exhibited by 48 (27.1%) PDAC patients and was significantly correlated with the higher expression of both α-SMA and CD31 mRNA, as well as patients’ T4 staging (Fig. 1e). Given the T4-staging tumor involves celiac arteries, its association with endothelial cells and EndoMT-related events is to be expected. Additionally, there is also a significant correlation between positive EndoMT index and positive M2-macrophage index (CD163^high^ and CD204^high^) in these 177 PDAC specimens (Fig. 1e). This association was also suggested by detecting M2-macrophages nearby EndoMT cells in the tissue specimens of our PDAC patients (Fig. 1f).

### Mouse model validates the association of M2-macrophages with EndoMT-involved PDAC

**Fig. 2:**
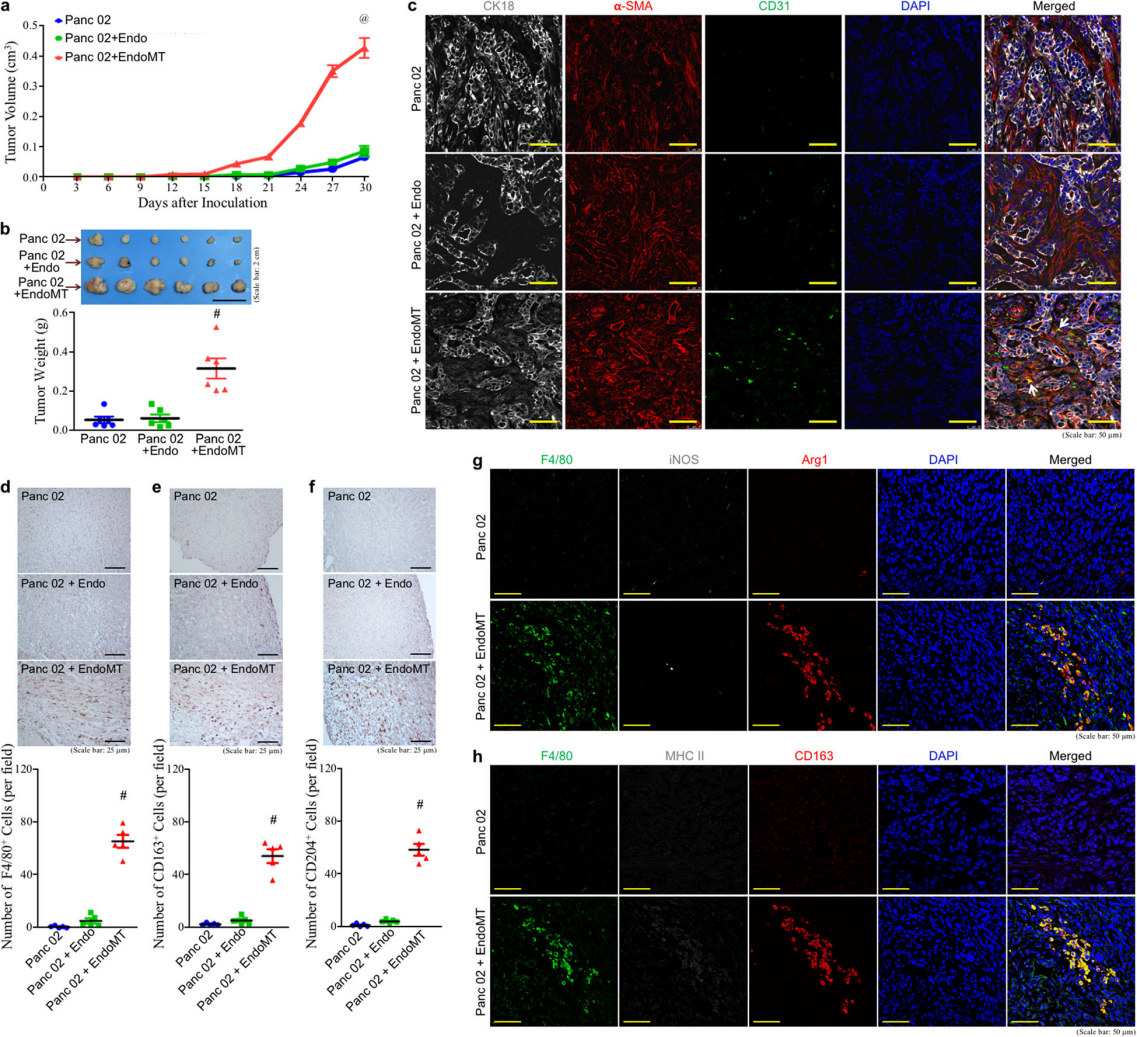
EndoMT-derived cells promote tumor formation with high macrophage infiltration. **a**, **b** Enhancing effect of EndoMT-derived cells on the tumor growth of Panc 02 cell grafts. C57BL/6 mice were subcutaneously injected with Panc 02 cells alone or together with endothelial cells (designated as “Endo”) or EndoMT-derived cells (designated as “EndoMT”) (*n* = 6 per group). The sizes of developing tumors were superficially measured using a Vernier caliper since day 3 post-inoculation with the formula of ½ × length × width^2^ (**a**). ^@^*P* < 0.001 when “Panc 02 + EndoMT” group was compared with “Panc 02” or “Panc 02 + Endo” group. Mice were sacrificed on day 30 post-inoculation and tumors were removed (**b**). ^#^*P* < 0.01 when “Panc 02 + EndoMT” group was compared with “Panc 02” or “Panc 02 + Endo” group. (**c**) The tumor masses in “Panc 02 + EndoMT” group comprised not only CK18-expressing Panc 02 cells and infiltrating α-SMA^+^ stromal cells, but also α-SMA^+^ and CD31^+^ EndoMT-derived cells (indicated by white arrows). **d–f** IHC of macrophages from the tumors formed by Panc 02 cells alone or Panc 02 cells plus endothelial cells or EndoMT-derived cells. Tissue sections were stained with F4/80 (**d**), CD163 (**e**), or CD204 (**f**) antibody to detect pan-macrophages (F4/80^+^ cells) or M2-type macrophages (CD163^+^ or CD204^+^ cells). ^#^*P* < 0.01 when “Panc 02 + EndoMT” group was compared with “Panc 02” or “Panc 02 + Endo” group. **g**, **h** IHF of the combination of F4/80, iNOS, and Arg1 (**g**) or the combination of F4/80, MHC II, and CD163 (**h**), confirming the infiltration of M2-type (F4/80^+^Arg1^+^ or F4/80^+^CD163^+^) macrophages into the tumors derived from Panc 02 plus EndoMT cells

To validate the association of M2-macrophages with EndoMT cells-involved PDAC, C57BL/6 mice were subcutaneously inoculated with mouse pancreatic cancer Panc 02 cells together with mouse enodothelial cells (3B-11 cells) or EndoMT cells (OPN-treated 3B-11 cells, Additional file 2: Fig. S1). The tumor-forming ability of Panc 02 plus EndoMT cells was significantly promoted when compared with that of Panc 02 cells alone or Panc 02 plus endothelial cells (Fig. 2a, b). These greatly promoted tumor masses comprised not only cytokeratin-18 (CK18)-expressing Panc 02 cells and infiltrating α-SMA^+^ stromal cells, but also EndoMT cells that highly expressed α-SMA and CD31, confirming the participation of EndoMT cells in the tumors (Fig. 2c). IHC analyses further reveal that such tumor masses contained comparable levels of F4/80^+^ cells (pan macrophages, Fig. 2d), CD163^+^ cells (M2-macrophages, Fig. 2e), and CD204^+^ cells (M2-macrophages, Fig. 2f), suggesting that EndoMT-mediated tumor promotion was associated with infiltrating M2-macrophages. IHF results showing F4/80^+^ macrophages expressed M2-type macrophage markers like arginase 1 (Arg1, Fig. 2 g) and CD163 (Fig. 2 h) also confirmed that M2 but not M1-macrophages predominated in the EndoMT cells-promoted tumor masses.

### EndoMT cells stimulate macrophage infiltration and M2-polarization

**Fig. 3:**
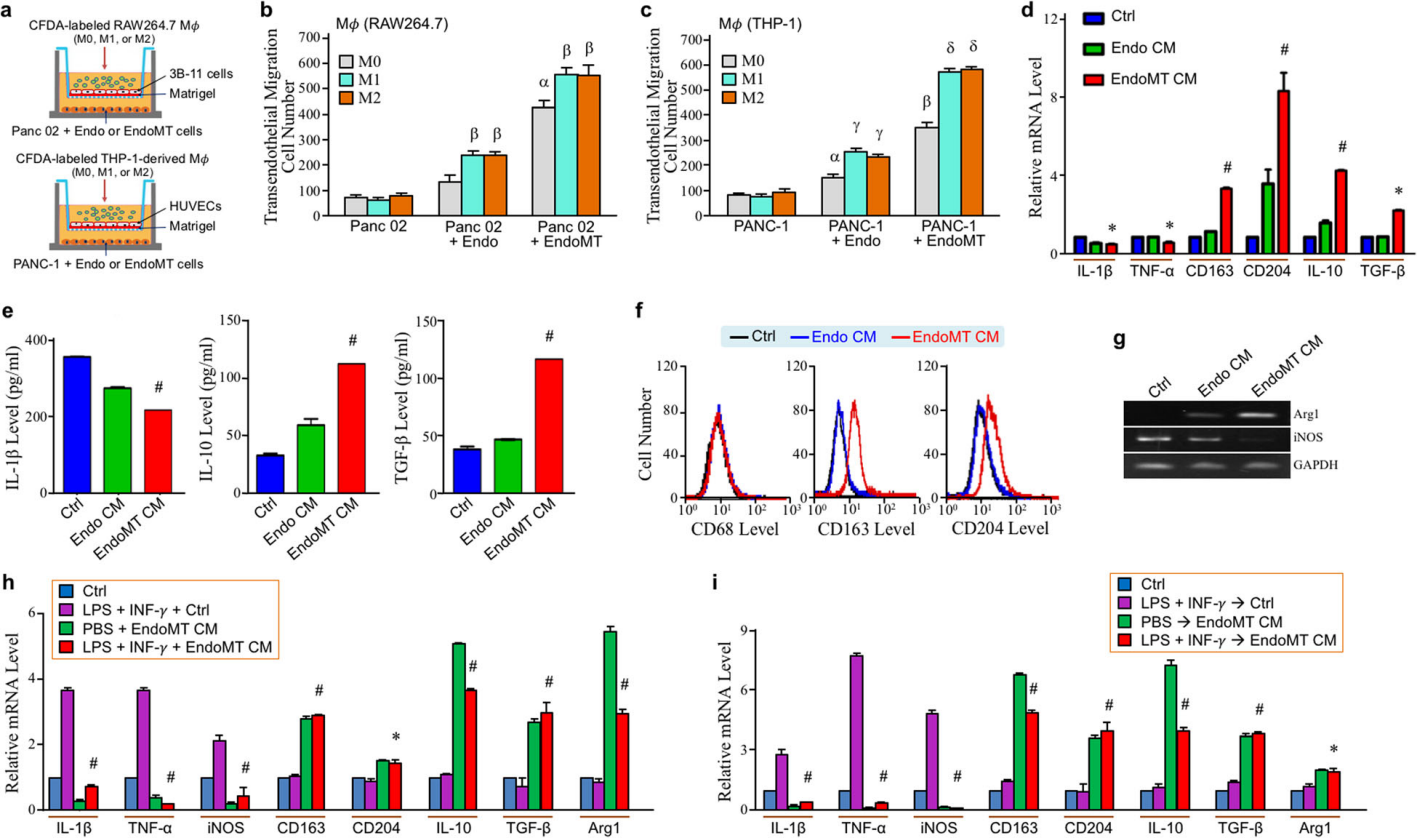
EndoMT-derived cells stimulate macrophage infiltration and M2-polarization. **a** Set-ups of transendothelial migration assays using mouse (upper panel) or human (bottom panel) cells. **b** Transendothelial migration activities of non-polarized, M1-polarized, and M2-polarized RAW264.7 cells upon co-culture with Panc 02 cells alone or Panc 02 cells plus 3B-11 cells or OPN-treated 3B-11 cells. ^α^*P* < 0.01 when compared with “Panc 02” group. ^β^*P* < 0.01 when compared with “M0” group. **c** Transendothelial migration activities of non-polarized, M1-polarized, and M2-polarized differentiated THP-1 cells upon co-culture with PANC-1 cells alone or PANC-1 cells plus HUVECs or OPN-treated HUVECs. ^α^*P* < 0.05 and ^β^*P* < 0.01 when compared with “PANC-1” group. ^γ^*P* < 0.05 and ^δ^*P* < 0.01 when compared with “M0” group. **d** mRNA levels of IL-1β, TNF-α, CD163, CD204, IL-10, and TGF-β in the THP-1-derived macrophages treated 24 h with control medium (Ctrl), Endo CM, or EndoMT CM. *P < 0.05 and ^#^*P* < 0.01 when compared with “Ctrl” group. **e** Secreted levels of IL-1β, IL-10, and TGF-β from the THP-1-derived macrophages treated 24 h with Ctrl, Endo CM, or EndoMT CM. THP-1-derived macrophages were pre-incubated with 1% FBS-containing RPMI 1640 medium for 16 h. The medium was then replaced with Ctrl, Endo CM, or EndoMT CM for further 24 h. The treated macrophages were further incubated with 5 ml of fresh 1% FBS-containing medium for 24 h. The media were finally collected for ELISAs. ^#^*P* < 0.01 when compared with “Ctrl” group. **f** Cell-surface levels of CD163 and CD204 in the THP-1-derived macrophages treated 24 h with Ctrl, Endo CM, or EndoMT CM. **g** mRNA expression status of Arg1 and iNOS in the THP-1-derived macrophages treated 24 h with Ctrl, Endo CM, or EndoMT CM. **h** mRNA levels of IL-1β, TNF-α, iNOS, CD163, CD204, IL-10, TGF-β, and Arg1 in the THP-1-derived macrophages treated 24 h with LPS plus IFN-γ (M1-inducer) in control medium (Ctrl) or EndoMT CM. *P < 0.05 and ^#^*P* < 0.01 when compared with “LPS + IFN-γ + Ctrl” group. **i** mRNA levels of IL-1β, TNF-α, iNOS, CD163, CD204, IL-10, TGF-β, and Arg1 in the THP-1-derived macrophages pretreated with LPS plus IFN-γ for 6 h and treated with control medium (Ctrl) or EndoMT CM containing fresh LPS and IFN-γ for further 24 h. *P < 0.05 and ^#^*P* < 0.01 when compared with “LPS + IFN-γ ➔ Ctrl” group

Next, we wondered if EndoMT cells exerted any effect on macrophage infiltration and M2-polarization. The transendothelial migration of macrophages towards PDAC cells either alone or in the presence of endothelial or EndoMT cells was investigated using Transwell^TM^ invasion assays depicted in Fig. 3a. Both mouse (Fig. 3b) and human (Fig. 3c) cell models revealed that not only the naive macrophages, higher numbers of M1- and M2-macrophages were recruited more by PDAC cells co-cultured with EndoMT cells compared with those with endothelial cells. To test if EndoMT cells secretion mediated macrophage M2-polarization, the CM of endothelial and EndoMT cells were collected to treat macrophages. mRNA levels of M1-associated IL-1β and tumor necrosis factor (TNF)-α were significantly downregulated whereas those of M2-associated CD163, CD204, IL-10, and TGF-β were significantly upregulated in the macrophages treated with EndoMT CM (Fig. 3d). Consistently, cellular secretion level of IL-1β was reduced but those of IL-10 and TGF-β were elevated (Fig. 3e). Flow cytometric analyses also revealed that EndoMT CM induced cell-surface levels of CD163 and CD204 on macrophages (Fig. 3f). As for metabolic markers, EndoMT CM induced M2-associated *Arg1* gene expression but suppressed M1-associated *inducible NO synthase* (*iNOS*) gene expression (Fig. 3 g). The suppressive effect of EndoMT CM on macrophage M1-polarization was further confirmed by treating macrophages with M1-inducer in EndoMT CM. Induction of IL-1β, TNF-α, and iNOS expressions by LPS and IFN-γ was drastically abolished by EndoMT CM (Fig. 3 h). However, EndoMT CM still caused significant increases of CD163, CD204, IL-10, TGF-β, and Arg1 mRNA levels under the presence of LPS and IFN-γ (Fig. 3h). Suppression of M1-polarization by EndoMT CM was also observed even when macrophages had been stimulated with LPS plus IFN-γ for 6 h (Fig. 3i). Together, these results suggest that EndoMT cells secreted some factor(s) to suppress macrophage M1-type activation but facilitate the polarization toward M2-type. Similar results were also obtained from the EndoMT cells induced by TGF-β instead of OPN (Additional file 2: Figure S2).

### EndoMT cells secrete HSP90α to induce macrophage M2-polarization

**Fig. 4:**
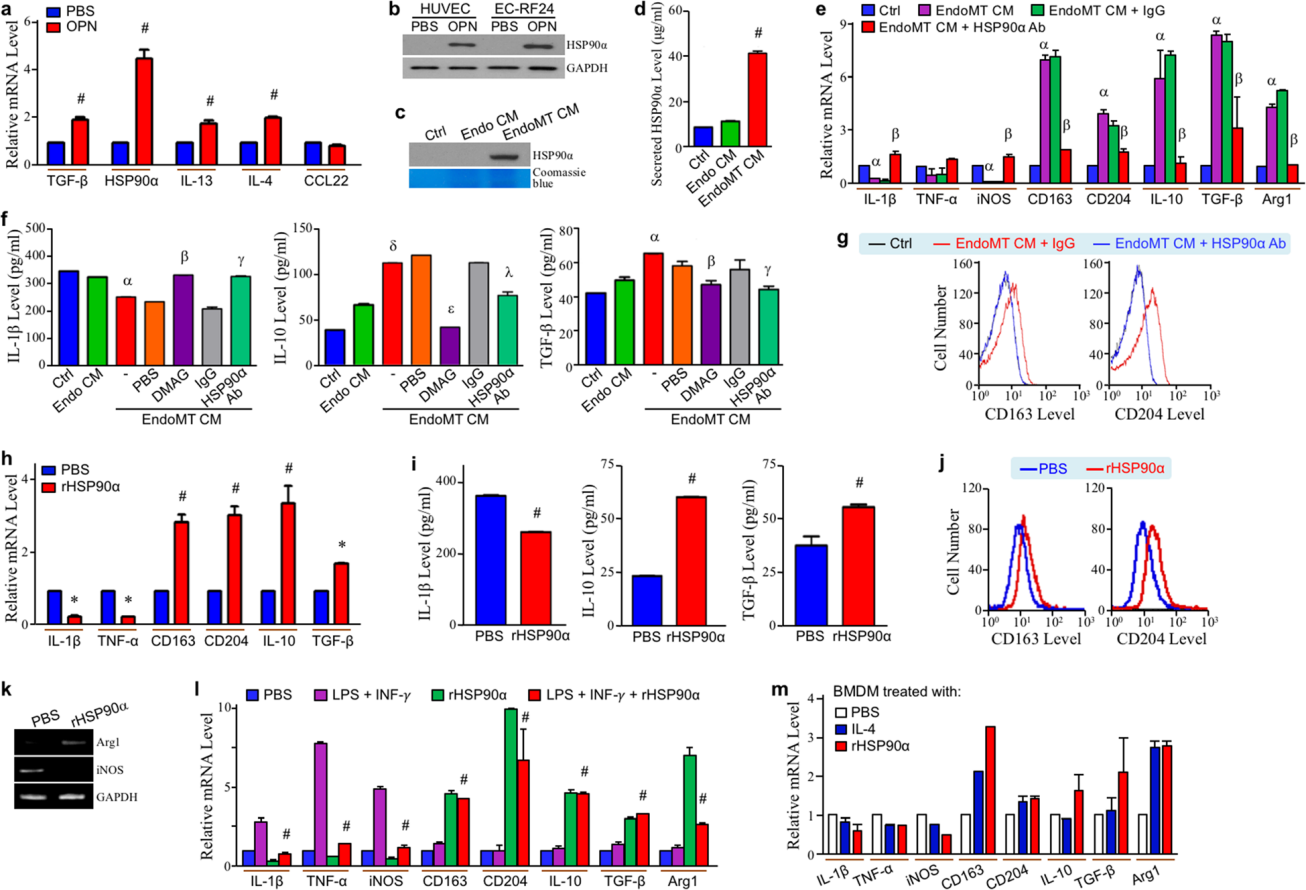
EndoMT-derived cells secrete HSP90α to induce macrophage M2-polarization. **a** mRNA levels of TGF-β, HSP90α, IL-13, IL-4, and C-C motif chemokine ligand 22 (CCL22) in EndoMT-derived cells. TGF-β, HSP90α, IL-13, and IL-4 mRNA expressions were significantly induced in the HUVECs treated 24 h with OPN. ^#^*P* < 0.01 when compared with PBS treatment. **b** HSP90α protein levels in the HUVECs and EC-RF24 cells treated 24 h with PBS or 0.3 μg/ml of OPN. HSP90α protein expression was obviously induced in OPN-treated endothelial cells. **c**, **d** Secreted HSP90α levels of HUVECs and their EndoMT derivatives. Ctrl, Endo CM, and EndoMT CM were prepared as described in Materials and Methods, and subjected to analyses of secreted HSP90α levels using immunoblot analysis (**c**) and ELISA (**d**). Induction of HSP90α secretion was significantly detected in OPN-treated HUVECs. ^#^*P* < 0.01 when compared with “Ctrl” or “Endo CM” group. **e** mRNA levels of IL-1β, TNF-α, iNOS, CD163, CD204, IL-10, TGF-β, and Arg1 in the THP-1-derived macrophages treated 24 h with control medium (Ctrl), EndoMT CM, or EndoMT CM plus control IgG or anti-HSP90α antibody. ^α^*P* < 0.05 when compared with “Ctrl” group. ^β^*P* < 0.05 when compared with “EndoMT CM + IgG” group. **f** Secreted levels of IL-1β, IL-10, and TGF-β from the THP-1-derived macrophages treated 24 h with Ctrl, Endo CM, or EndoMT CM in the absence or presence of PBS, 1 μM DMAG-N-oxide, or 10 μg/ml of IgG or anti-HSP90α antibody. THP-1-derived macrophages were pre-incubated with 1% FBS-containing medium for 16 h and subjected to indicated treatments for further 24 h. The treated macrophages were then incubated with 5 ml of fresh 1% FBS-containing medium for 24 h. The media were finally collected for ELISAs. ^α^*P* < 0.05 and ^δ^*P* < 0.01 when compared with “Ctrl” group. ^β^*P* < 0.05 and ^ε^*P* < 0.01 when compared with “EndoMT CM + PBS” group. ^γ^*P* < 0.05 and ^λ^*P* < 0.01 when compared with “EndoMT CM + IgG” group. **g** Cell-surface levels of CD163 and CD204 in the THP-1-derived macrophages treated 24 h with Ctrl or EndoMT CM plus control IgG or anti-HSP90α antibody. **h** mRNA levels of IL-1β, TNF-α, CD163, CD204, IL-10, and TGF-β in the THP-1-derived macrophages treated 24 h with PBS or 15 μg/ml of rHSP90α. *P < 0.05 and ^#^*P* < 0.01 when compared with PBS treatment. **i** Secreted levels of IL-1β, IL-10, and TGF-β from the THP-1-derived macrophages treated 24 h with PBS or 15 μg/ml of rHSP90α. THP-1-derived macrophages were pre-incubated with 1% FBS-containing medium for 16 h and then added with PBS or 15 μg/ml of rHSP90α for another 24 h. The treated macrophages were further incubated with 5 ml of fresh 1% FBS-containing medium for 24 h. The media were finally collected for ELISAs. ^#^*P* < 0.01 when compared with PBS treatment. **j** Cell-surface levels of CD163 and CD204 in the THP-1-derived macrophages treated 24 h with PBS or 15 μg/ml of rHSP90α. **k** mRNA expression status of Arg1 and iNOS in the THP-1-derived macrophages treated 24 h with PBS or 15 μg/ml of rHSP90α. **l** mRNA levels of IL-1β, TNF-α, iNOS, CD163, CD204, IL-10, TGF-β, and Arg1 in the THP-1-derived macrophages treated 24 h with LPS plus IFN-γ in the absence or presence of rHSP90α. ^#^*P* < 0.01 when compared with “LPS + IFN-γ” group. **m** mRNA levels of IL-1β, TNF-α, iNOS, CD163, CD204, IL-10, TGF-β, and Arg1 in the mouse BMDMs treated 24 h with PBS, 20 ng/ml of IL-4, or 15 μg/ml of rHSP90α. rHSP90α significantly induced M2-polarization of BMDMs

Given that TGF-β, IL-4, and IL-13 are known inducers of macrophage M2-polarization, their mRNA expressions were all upregulated in OPN-treated endothelial cells (Fig. 4a). Notably, HSP90α mRNA was induced even more in the EndoMT cells (Fig. 4a). HSP90α protein levels were also upregulated both in the intracellular and secreted fractions (Fig. 4b–d). To investigate whether the secreted HSP90α was involved in EndoMT-associated macrophage M2-polarization, we treated macrophages with EndoMT CM in the presence of anti-HSP90α antibody. Downregulation of IL-1β, TNF-α, and iNOS mRNA expressions and upregulation of CD163, CD204, IL-10, TGF-β, and Arg1 mRNA levels were effectively restored by HSP90α antibody (Fig. 4e). Consistently, reduction of IL-1β secretion and induction of IL-10 and TGF-β secretions were both drastically antagonized by the eHSP90α inhibitor DMAG-N-oxide and anti-HSP90α antibody (Fig. 4f). Flow cytometric analyses also showed that HSP90α antibody drastically abolished EndoMT CM-induced macrophage CD163 and CD204 levels (Fig. 4g). Next, we treated macrophages with purified recombinant HSP90α (rHSP90α). As shown in Fig. 4h, mRNA levels of IL-1β and TNF-α were downregulated but those of CD163, CD204, IL-10, and TGF-β were upregulated in these rHSP90α-treated cells. Cellular IL-1β secretion was reduced whereas the secretion levels of IL-10 and TGF-β were increased (Fig. 4i). rHSP90α also induced M2-associated cell-surface markers CD163 and CD204 (Fig. 4j) as well as metabolic marker *Arg1* gene expression (Fig. 4 k). Besides, rHSP90α suppressed the M1-polarization induced by LPS plus IFN-γ (Fig. 4 l). Taken together, our results suggest that HSP90α secreted from EndoMT cells inhibited macrophage M1-type activation and exerted a stimulatory effect on M2-polarization. The result was consistent using the BMDMs treated with rHSP90α (Fig. 4 m).

### HSP90α secretion is amplified simultaneously with macrophage M2-polarization

**Fig. 5:**
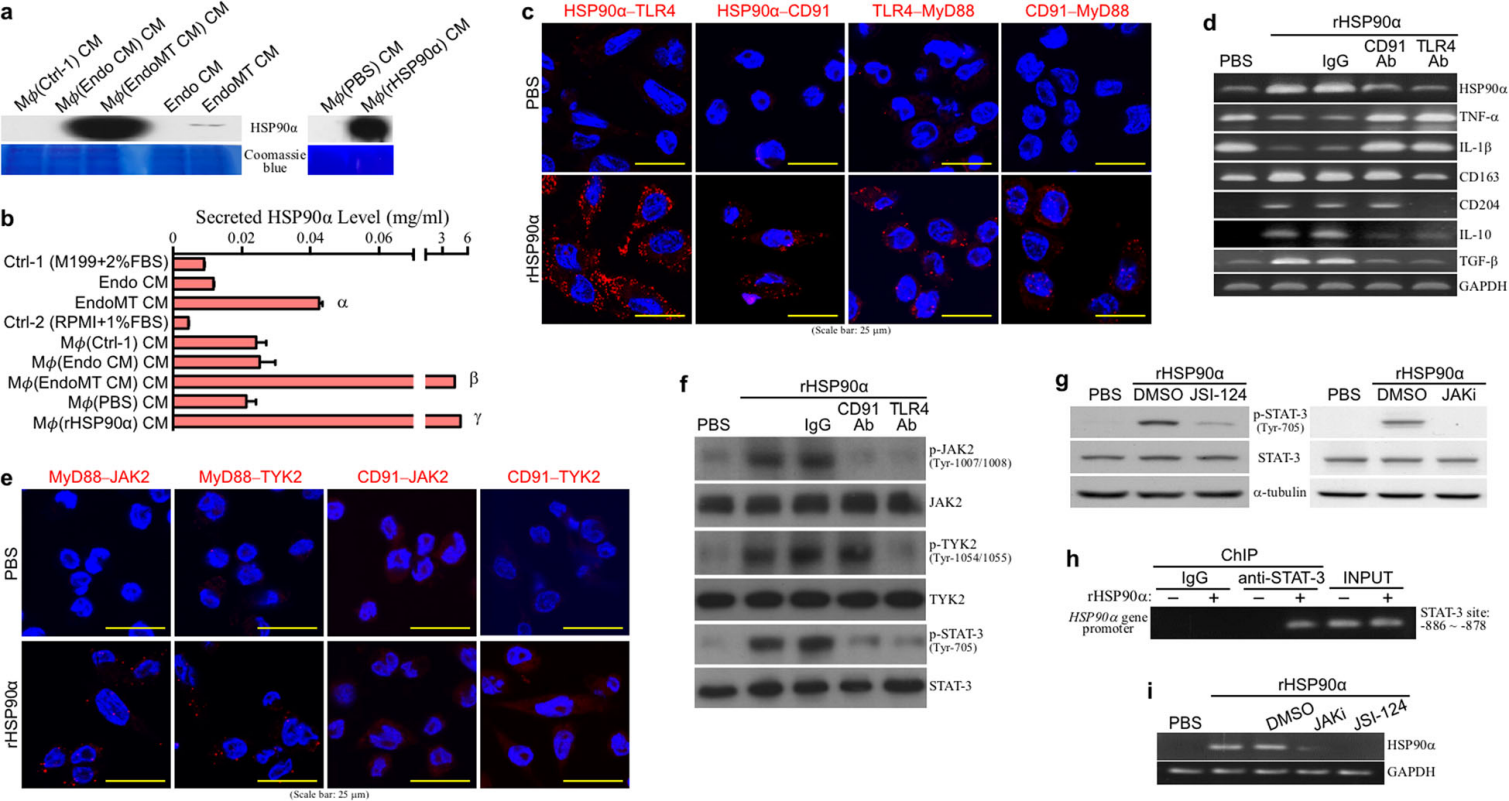
eHSP90α induces a feedforward loop of macrophage HSP90α secretion. **a**, **b** Abundant HSP90α secretion from the THP-1-derived macrophages after EndoMT CM and rHSP90α stimulation. THP-1-derived macrophages were pre-incubated with 1% FBS-containing medium for 16 h. The medium was then added with PBS or 15 μg/ml of rHSP90α, or replaced with Ctrl, Endo CM, or EndoMT CM for another 24 h. The treated macrophages were further incubated with 5 ml of fresh 1% FBS-containing medium for 24 h. The media were finally collected and subjected to immunoblot analysis (**a**) and quantitative measurement (**b**) of HSP90α. ^α^*P* < 0.05 when compared with “Ctrl-1” group. ^β^*P* < 0.05 when compared with “Mø(Ctrl-1) CM” group. ^γ^*P* < 0.05 when compared with “Mø(PBS) CM” group. **c** eHSP90α binds macrophage TLR4 and CD91 receptors. PLAs showed red fluorescent dots in rHSP90α-treated macrophages by using the antibody combinations detecting the physical interactions of TLR4–HSP90α, CD91–HSP90α, TLR4–MyD88, and CD91–MyD88, suggesting that HSP90α binds to TLR4 and CD91 which could further recruit MyD88. **d** mRNA levels of HSP90α, TNF-α, IL-1β, CD163, CD204, IL-10, and TGF-β in the macrophages treated with PBS or rHSP90α in the absence or presence of control IgG or the antibody against CD91 or TLR4. Phenomena induced by rHSP90α such as downregulation of TNF-α and IL-1β mRNA levels and upregulation of HSP90α, IL-10, and TGF-β mRNA level were drastically abolished by CD91 and TLR4-antagonizing antibodies, whereas rHSP90α-induced CD163 and CD204 mRNA levels were repressed by the antibody antagonizing TLR4 but not CD91. **e** eHSP90α induces associations of MyD88 with JAK2 and TYK2. PLAs showed red fluorescent dots in rHSP90α-treated macrophages when using the antibody combinations detecting the interactions of MyD88–JAK2 and MyD88–TYK2 but not the antibody combinations detecting CD91–JAK2 and CD91–TYK2 interactions, suggesting that eHSP90α induced physical associations of JAK2 and TYK2 with MyD88 but not CD91. **f** Levels of phosphorylated/activated JAK2, TYK2, and STAT-3 in the macrophages treated with PBS or rHSP90α in the absence or presence of control IgG or anti-CD91 or -TLR4 antibody. Phosphorylation/activation of JAK2, TYK2, and STAT-3 was detected in rHSP90α-treated macrophages. Both TLR4 and CD91-antagonizing antibodies could inhibit the phosphorylation of JAK2 and STAT-3. However, anti-TLR4 antibody but not anti-CD91 antibody inhibited rHSP90α-induced TKY2 phosphorylation. **g** Levels of phosphorylated/activated STAT-3 in the macrophages treated with PBS or rHSP90α in the absence or presence of 10 μM JAK2/TYK2 inhibitor (JSI-124) or 10 nM JAK2 inhibitor (JAKi). **h** ChIP assay showed that rHSP90α induced binding of STAT-3 to *HSP90α* gene promoter in macrophages. **i** mRNA level of HSP90α in the macrophages treated with PBS or rHSP90α in the absence or presence of JSI-124 or JAKi. rHSP90α-induced macrophage HSP90α expression was effectively prevented by JSI-124 and JAKi

Besides M2-polarization, EndoMT CM or rHSP90α-treated macrophages exhibited amplitude of HSP90α secretion (Fig. 5a). Approximately 4.5 mg/ml and 5.1 mg/ml of HSP90α were detected from the CM of the macrophages pretreated with EndoMT CM and rHSP90α, respectively, as against ~ 24.3 μg/ml detected from those of control macrophages (Fig. 5b). We wondered if eHSP90α exerted a stimulatory effect on macrophage HSP90α expression. By PLA, physical associations of HSP90α with cell receptors TLR4 and CD91 as well as the recruitment of downstream MyD88 were detected in rHSP90α-treated macrophages (Fig. 5c). The physical association between CD91 and TLR4 was also enhanced upon rHSP90α treatment (Additional file 2: Figure S3). Despite the fact that rHSP90α-induced CD163 and CD204 mRNA expressions were repressed by the antibody antagonizing TLR4 but not CD91, both downregulation of TNF-α and IL-1β mRNA levels and upregulation of HSP90α, IL-10, and TGF-β mRNA expressions were drastically abolished by TLR4 and CD91-antagonizing antibodies (Fig. 5d), suggesting that eHSP90α induced macrophage HSP90α expression and M2-polarization by acting through TLR4 and CD91. Our further results revealed that the known downstream kinases JAK2 and TYK2 were recruited onto MyD88 upon rHSP90α stimulation (Fig. 5e). Phosphorylation of JAK2 and TYK2 was also detected in rHSP90α-treated macrophages, while the presence of TLR4 or CD91-antagonizing antibody effectively prevented such phenomenon (Fig. 5f). rHSP90α-induced STAT-3 phosphorylation was also efficiently blocked by CD91 or TLR4-antagonizing antibody as well as by inhibitors targeting JAK2 or both JAK2 and TYK2 (Fig. 5f, g), suggesting that STAT-3 was a downstream transcription factor of the TLR4/CD91–MyD88–JAK2/TYK2 pathway. A putative STAT-3-binding site was recognized on the promoter region of *HSP90α* gene. It was confirmed by ChIP assay showing that rHSP90α induced STAT-3 binding to the site of *HSP90α* gene promoter (Fig. 5 h). The inhibitors targeting JAK2/TYK2–STAT-3 signaling axis also repressed rHSP90α-induced HSP90α mRNA expression in macrophages (Fig. 5i), confirming that the signaling cascade is indeed involved in eHSP90α-stimulated macrophage HSP90α expression.

### Anti-HSP90α antibody exhibits potent therapeutic efficacy in EndoMT cells-promoted cancer

**Fig. 6:**
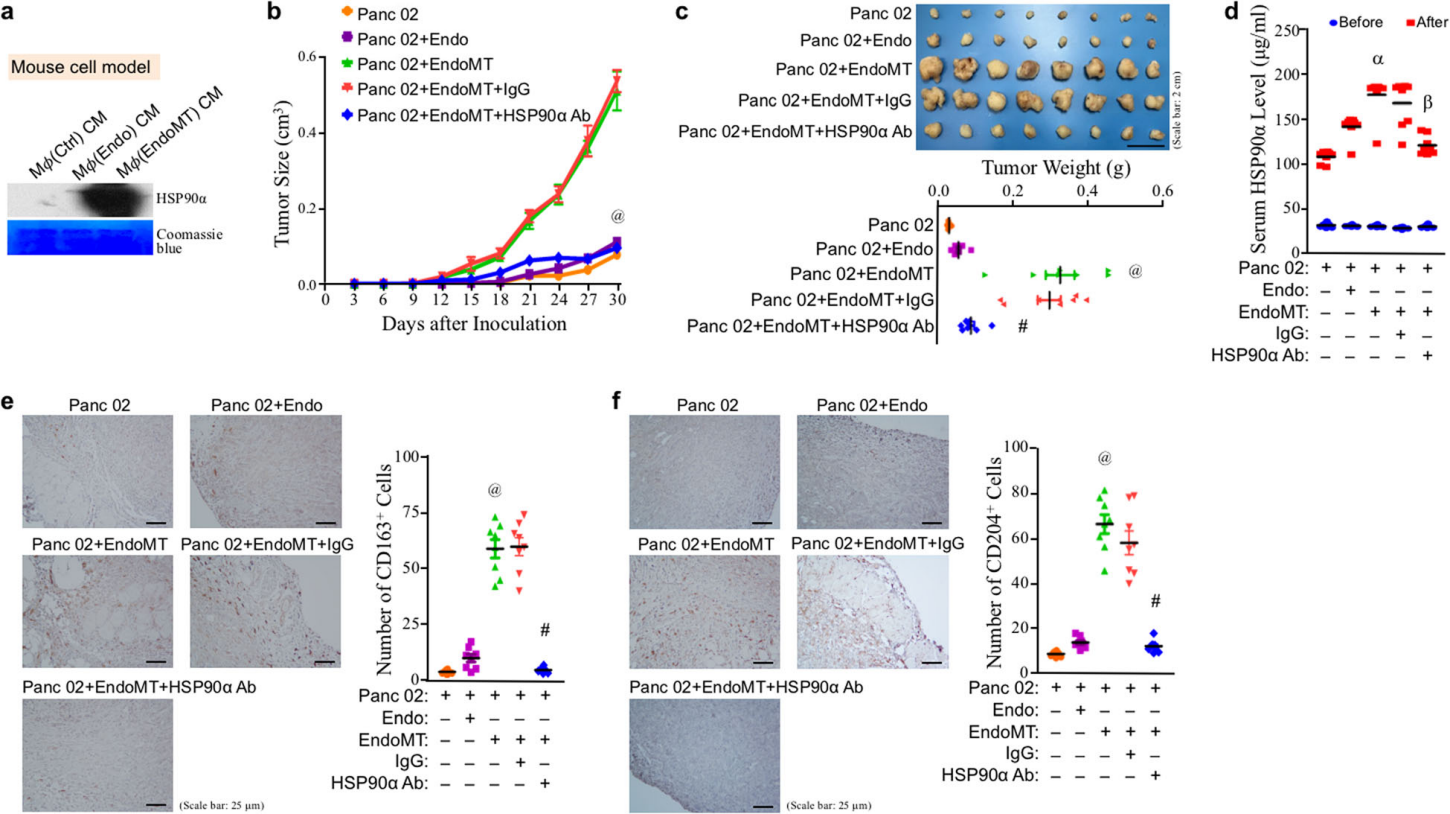
Anti-HSP90α antibody potently inhibits EndoMT-promoted and macrophage-associated tumor growth. **a** Abundant HSP90α secretion from the mouse macrophages after EndoMT CM stimulation. RAW264.7 cells were pre-incubated with 1% FBS-containing medium for 16 h. The medium was then replaced with Ctrl, Endo CM, or EndoMT CM for another 24 h. The treated macrophages were further incubated with 5 ml of fresh 1% FBS-containing medium for 24 h. The media were finally collected and subjected to immunoblot analysis. **b**, **c** Inhibitory effect of anti-HSP90α antibody on the tumor growth of Panc 02 cells plus EndoMT-derived cells. C57BL/6 mice were subcutaneously injected with Panc 02 cells alone or together with endothelial cells (designated as “Endo”) or EndoMT-derived cells (designated as “EndoMT”) (*n* = 8 per group). The mice inoculated with Panc 02 cells plus EndoMT-derived cells were further intravenously injected with control IgG or anti-HSP90α antibody (5 μg per g of body weight for each dosage) on day 4 post-inoculation. The injections were performed every 3 days for a period of 24 days. The sizes of developing tumors were superficially measured using a Vernier caliper since day 3 post-inoculation with the formula of ½ × length × width^2^ (**b**). ^@^*P* < 0.001 when “Panc 02 + EndoMT + HSP90α Ab” group was compared with “Panc 02 + EndoMT + IgG” group. Mice were sacrificed on day 30 post-inoculation and tumors were removed for weighing (**c**). ^@^*P* < 0.001 when compared with “Panc 02” group. ^#^*P* < 0.01 when compared with “Panc 02 + EndoMT + IgG” group. **d** Inhibitory effect of anti-HSP90α antibody on the elevation of serum HSP90α levels in the mice inoculated with Panc 02 cells plus EndoMT-derived cells as described above. Serum samples were collected from the mice on day 0 before cell-graft inoculation and on day 29 post-inoculation for HSP90α measurement by ELISA. ^α^*P* < 0.001 when compared with “Panc 02” group. ^β^*P* < 0.001 when compared with “Panc 02 + EndoMT + IgG” group. **e**, **f** Reduction of M2-type macrophage levels by anti-HSP90α antibody in the tumors derived from Panc 02 cells plus EndoMT-derived cells. Tumor tissue sections of the mice treated as described above were immunohistochemically staining with anti-CD163 (**e**) or anti-CD204 (**f**) antibody. ^@^*P* < 0.001 when compared with “Panc 02” group. ^#^*P* < 0.001 when compared with “Panc 02 + EndoMT + IgG” group

A burst of HSP90α secretion was also detected from mouse macrophages pretreated with mouse EndoMT CM (Fig. 6a). To verify the involvement of secreted HSP90α in EndoMT cells-facilitated macrophage M2-polarization and tumor growth, the mice pre-inoculated with Panc 02 cells plus EndoMT cells were further intravenously administered with control IgG or anti-HSP90α antibody. The enhancement of tumor growth by EndoMT cells was drastically abolished by anti-HSP90α antibody (Fig. 6b, c). Interestingly, the elevation of serum HSP90α levels in the mice inoculated with Panc 02 cells plus EndoMT cells was also effectively suppressed after anti-HSP90α therapy (Fig. 6d). Consistently, EndoMT cells-facilitated recruitment of CD163^+^ or CD204^+^ cells was also drastically suppressed by anti-HSP90α antibody (Fig. 6e, f). These results provide in vivo evidence for the role of eHSP90α in M2-macrophage-associated tumor promotion.

## Discussion

Macrophages and CAFs are two most common stromal cells in solid tumors [4, 25, 26]. A clinical correlation between the levels of CAFs and M2-macrophages has been revealed in CRC [22]. However, the relationship between EndoMT-derived CAFs and M2-macrophages in PDAC remains to be investigated. To study the clinical implications of EndoMT, we first explored a hallmark evaluating tumor EndoMT levels of PDAC patients’ specimens. Immunohistofluorescent staining assay has a limitation of quantifying the α-SMA^+^ and CD31^+^ EndoMT cells detected in PDAC tissues. We therefore searched for a molecular signature based on the publicized RNA expression data of the TCGA database. The mRNA expression profile of α-SMA in combination with fibroblast-specific protein 1 or/and fibroblast activation protein was excluded because these markers are not specific enough for EndoMT cells as they can also be induced in the cancer cells undergoing epithelial-to-mesenchymal transition (EMT). Instead, we proposed a potential EndoMT index according to the expression status of 3 lncRNAs LOC340340, LOC101927256, and MNX1-AS1. They were simultaneously downregulated in EndoMT cells, but highly expressed in other cell types such as PDAC cells and macrophages. To address whether these 3 lncRNAs could be downregulated in cancer cells or macrophages after interacting with EndoMT cells, we assayed the levels of the 3 lncRNAs from EndoMT CM-treated cancer cells and macrophages. Our preliminary data revealed that the 3 lncRNAs were upregulated in EndoMT CM-treated cancer cells. However, in EndoMT CM-treated macrophages, MNX1-AS1 and LOC101927256 were upregulated but LOC340340 was downregulated. Recently, overexpression of MNX1-AS1 has been reported to correlate with poor prognoses of epithelial ovarian cancer and gastric carcinoma [27, 28]. Our analysis of TCGA dataset also indicates that PDAC patients with higher MNX1-AS1 expression levels have a poorer prognosis (> mean vs. < mean, *P* = 0.038). Moreover, knockdown of MNX1-AS1 expression decreased cellular EMT, migration, and invasion in gastric carcinoma, breast cancer, and glioblastoma [28–30]. Unlike MNX1-AS1, LOC340340 and LOC101927256 are not yet well reported to associate with cancer. LOC340340 was mentioned as a VEGF-inducible lncRNA in endothelial cells [31]. Our TCGA analysis reveals that higher LOC340340 expression is also correlated with the poor prognosis of PDAC patients (> mean vs. < mean, *P* = 0.011).

Using the proposed EndoMT index, we have observed that PDAC tissues with positive EndoMT index are correlated with patients’ T4-staging and significantly exhibit positive M2-macrophage index. PDAC patients with T4-staging tumors are almost unresectable and have a 5-year survival rate as low as 3%. Although the sample size of T4 (*n* = 3) is too small to strongly demonstrate the statistical correlation between T4 staging and EndoMT index, the T4-staging tumors are celiac artery-involving tumors so as that they could have higher levels of endothelial cells and EndoMT cells. The correlation of EndoMT level with M2-macrophage level in PDAC tissues was confirmed using a mouse model of EndoMT-derived CAFs-involved PDAC, in which EndoMT-derived CAFs benefited the growth of PDAC cell grafts and the infiltration of M2-macrophages. Despite a clinical correlation between the levels of CAFs and M2-macrophages has been shown in CRC [22], further studies on the underlying mechanisms and therapeutic implications are still lacking. In co-culture cell model, transendothelial migration of M1 and M2-macrophages was highly stimulated by PDAC cells mixed with EndoMT-derived CAFs, compared with PDAC cells alone or PDAC cells plus endothelial cells. Moreover, EndoMT-derived CAFs were able to induce alternative activation of macrophage, M2-polarization. Besides the three known M2-polarization inducers TGF-β, IL-4, and IL-13, higher level of HSP90α was also expressed and secreted by EndoMT-derived CAFs to induce macrophage M2-polarization. This effect exerted by eHSP90α is distinct from that by intracellular HSP90α. The involvement of intracellular HSP90α in macrophage activation (M1-polarization) was first suggested based on the observation that the HSP90α inhibitor, geldanamycin, blocked Taxol or LPS-induced NF-κB activation and TNF-α expression in macrophages [32]. In interferon-γ-treated cancer cells, cytoplasmic HSP90α acts as a chaperone protecting JAK1/2 from degradation and thus enhances STAT-1 phosphorylation and the downstream gene expressions [33]. As regards eHSP90α, it can bind with TLR4 and CD91 of macrophages, and its induction of macrophage M2-polarization can be antagonized by anti-TLR4 or CD91 antibody. CD91 is an eHSP90α receptor on cancer cells and fibroblasts [17, 34], while TLR4 can associate with HSP90α in LPS-stimulated macrophages [35]. Both TLR4 and CD91 can function as the receptor/co-receptor for eHSP90α to induce macrophage M2-polarization. Interestingly, TLR4 is also the receptor for LPS to induce M1-type macrophages [36, 37], while CD91 negatively regulates LPS-induced M1-polarization since CD91-deleting macrophages expressed higher levels of TNF-α, IL-1β, and IL-6 with concomitant suppressions of efferocytosis and PI3K/Akt signaling [38, 39]. Therefore, the EndoMT-derived CAFs in tumor microenvironment can not only facilitate the infiltration of macrophages regardless of M0, M1, or M2-type, but also secrete HSP90α to induce CD91-mediated M2-polarization of the infiltrating macrophages.

The M2-type polarization is a generally occurring event for tumor-associated macrophages in response to multiple microenvironmental factors derived from tumor cells, stromal cells, and tissue stress [40]. M2-polarized macrophages do not only function as a key immunosuppressor in tumors, but also exacerbate cancer progression by promoting tumor angiogenesis and tumor cell EMT, migration, invasion, intravasation, survival in the circulation, and extravasation [41]. Higher levels of M2-macrophages have been clinically correlated with many human cancers and therefore considered to be used as diagnostic and prognostic markers [40, 42, 43]. Moreover, many therapeutic approaches targeting M2-macrophages by impairing infiltration and M2-polarization of macrophages are also undergoing and provide a direction to develop novel therapeutic strategies [41]. Our present paper is the first report demonstrating that eHSP90α secreted by EndoMT-derived CAFs is able to induce macrophage M2-polarization. Besides the M2-type markers, a feedforward loop was induced by eHSP90α and thus a large amount of HSP90α was expressed and secreted from eHSP90α-treated macrophages. Elevated HSP90α secretion has been known as an inflammation and stress-related event. HSP90α can be secreted by keratinocytes and fibroblasts associated with wound healing [14], as well as by cancer cells under unfavorable microenvironments to expedite cancer cell metastasis [16, 17]. It can also be secreted by EndoMT cells to exacerbate CRC via the induction of CRC cell stemness [12]. Additionally, HSP90α can be secreted by pancreas-infiltrating myeloid-derived macrophages and the stimulated pancreatic ductal epithelial cells to facilitate the macrophage-associated PDAC development [18]. Therefore, EndoMT-induced macrophage infiltration and M2-polarization result not only in well-known M2-macrophages-associated tumor-promoting effects (e.g., tumor immunosuppression and angiogenesis), but also create an eHSP90α-rich microenvironment to enhance PDAC tumor growth and malignant progression. Considering eHSP90α as therapeutic target, anti-HSP90α antibodies and the eHSP90α inhibitor DMAG-N-oxide have been tested for anti-cancer efficacies in mouse models recently [18, 44]. In our present study, anti-HSP90α antibody has shown a potent inhibitory efficacy against the EndoMT cells-facilitated M2-macrophage recruitment and PDAC tumor growth.

**Fig. 7:**
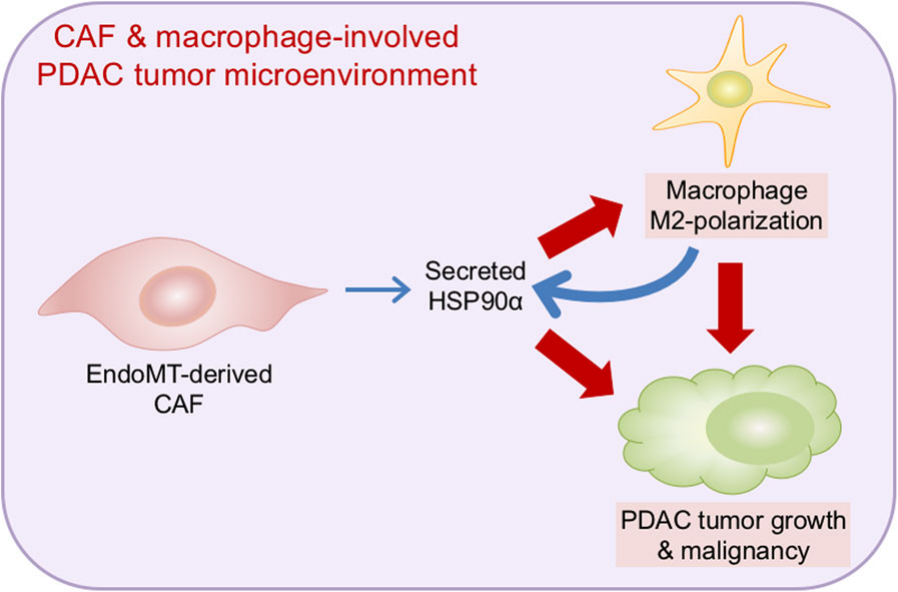
A schematic illustration summarizing our finding that EndoMT-derived CAFs can secrete HSP90α to induce macrophage M2-polarization and more HSP90α production to exacerbate PDAC tumor

In summary, the EndoMT level of PDAC tissues is significantly correlated with patients’ T4-staging and tumor M2-macrophage level. EndoMT-derived CAFs can secrete HSP90α to induce macrophage M2-polarization and more HSP90α production (Fig. 7). Anti-HSP90α antibody can exhibit a potent inhibitory efficacy against the EndoMT and M2-macrophage-involved PDAC tumor growth.

## Conclusions

CAFs contribute to desmoplasia of many malignancies including PDAC and play roles in promoting tumor growth, immunosuppression, and malignant progression. Targeting CAFs and the related events is a challenging field which can inspire novel strategies to improve PDAC therapeutics. Considering CAFs can arise from the EndoMT of endothelial cells, we herein investigated the clinical relevance of EndoMT in PDAC and explored its underlying mechanism and therapeutic implication. Our study reveals that EndoMT-derived cells can secrete HSP90α to harness HSP90α-overproducing M2-type macrophages to promote PDAC tumor growth, and such effect can be targeted and abolished by anti-HSP90α antibody. Anti-HSP90α antibody can be developed as a potent therapeutic agent against the EndoMT-promoted and M2-macrophage-involved PDAC tumor.

## Supplementary information

**Additional file 1: Table S1.** List of the antibodies used in this study. **Table S2.** The primers and PCR conditions adopted in this study. All reactions started at 95°C for 5 min and terminated at 72°C for 7 min.

**Additional file 2: Figure S1.** OPN induces EndoMT. **A,** mRNA levels of VE-cadherin, CD31, and α-SMA in the HUVECs pre-incubated 16 h with 2% FBS-containing M199 medium and then added with PBS or 0.3 μg/ml of OPN for further 24 h. We have previously reported that OPN induced EndoMT of HUVECs and immortalized endothelial cell line EC-RF24 (12). Consistently, the present data shows that OPN induced down-regulation of endothelial cell marker genes (VE-cadherin and CD31) but up-regulation of mesenchymal cell marker gene α-SMA. **B,** mRNA levels of VE-cadherin, Tie1, Tie2, CD31, α-SMA, and fibronectin in mouse immortalized endothelial cell line 3B-11 pre-incubated 16 h with 1% FBS-containing RPMI 1640 medium and then added with PBS or 0.3 μg/ml of OPN for further 24 h. The data revealed that OPN also induced EndoMT of 3B-11 cells. **Figure S2.** Using TGF-β-induced EndoMT model to confirm EndoMT CM-induced marophage M2-polarization. EndoMT CM and control medium (CTRL) were prepared as described in the Methods section except 20 ng/ml of TGF-β was used instead of OPN. THP-1-derived macrophages were treated with CTRL or EndoMT CM for 24 h. Relative mRNA levels of IL-1β, TNF-α, iNOS, CD163, CD204, IL-10, TGF-β, and Arg1 were assessed by qPCR analyses. #, *P* < 0.001 when compared with CTRL. **Figure S3.** eHSP90α enhances the physical association of CD91 with TLR4. PLAs showed red fluorescent dots in PBS or rHSP90α-treated macrophages by using the antibody combination detecting the physical interaction of CD91–TLR4. The level of red fluorescent dots was increased upon rHSP90α treatment.

## Data Availability

All data generated or analyzed during this study are included in this article and its additional files.

## Abbreviations

Arg1: Arginase 1
α-SMA: α-Smooth muscle actin
BMDM: Bone marrow-derived macrophage
CAF: Cancer-associated fibroblast
ChIP: Chromatin immunoprecipitation
CM: Conditioned medium
CRC: Colorectal cancer
EndoMT: Endothelial-to-mesenchymal transition
eHSP90α: Extracellular HSP90α
HUVEC: Human umbilical vein endothelial cell
IL: Interleukin
iNOS: Inducible NO synthase
lncRNA: Long non-coding RNA
LPS: Lipopolysaccharide
OPN: Osteopontin
PDAC: Pancreatic ductal adenocarcinoma
PLA: Proximity ligation assay
rHSP90α: Recombinant HSP90α
TGF-β: Transforming growth factor-β
TNF-α: Tumor necrosis factor-α
TPA: 12-*O*-tetradecanoyl-13-phorbol acetate
