# Supplementary material for: Endothelial-mesenchymal transition harnesses HSP90α-secreting M2-macrophages to exacerbate pancreatic ductal adenocarcinoma
Source: J Hematol Oncol. 2019 Dec 17;12:138. doi: 10.1186/s13045-019-0826-2 (PMC6918594; doi:10.1186/s13045-019-0826-2)
Supplement: Supplementary file 1 — Additional file 1: Table S1. List of the antibodies used in this study. Table S2. The primers and PCR conditions adopted in this study. All reactions started at 95°C for 5 min and terminated at 72°C for 7 min. [file 13045_2019_826_MOESM1_ESM.docx]

**Additional File 1**

**Table S1.** List of the antibodies used in this study

| Experiment | Antibody | Company | Catalog No. | Titer |
| --- | --- | --- | --- | --- |
| IHF | rabbit anti-α-SMA | Abcam, Cambridge, UK | ab32575 | 1:100 |
|  | goat anti-CD31 | Santa Cruz Biotechnology, Santa Cruz, CA | sc-1505 | 1:50 |
|  | mouse anti-CD163 | Santa Cruz Biotechnology, Santa Cruz, CA | sc-20066 | 1:50 |
|  | mouse anti-CK18 | Abcam, Cambridge, UK | ab668 | 1:100 |
|  | rat anti-F4/80 | Abcam, Cambridge, UK | ab6640 | 1:100 |
|  | rabbit anti-iNOS | GeneTex Inc., Hsinchu City, Taiwan | GTX15323 | 1:100 |
|  | mouse anti-Arg1 | Santa Cruz Biotechnology, Santa Cruz, CA | sc-271430 | 1:80 |
|  | rabbit anti-MHC II | Abcam, Cambridge, UK | ab180779 | 1:100 |
|  | anti-rabbit IgG-Alexa Fluor 568 | Thermo Fisher Scientific, Waltham, MA | A10042 | 1:500 |
|  | anti-goat IgG-Alexa Fluor 647 | Thermo Fisher Scientific, Waltham, MA | A21447 | 1:500 |
|  | anti-mouse IgG-Alexa Fluor 488 | Thermo Fisher Scientific, Waltham, MA | A21202 | 1:500 |
|  | anti-rat IgG-Alexa Fluor 488 | Thermo Fisher Scientific, Waltham, MA | A21208 | 1:500 |
| IHC | rat anti-F4/80 | AbD Serotec, Raleigh, NC | MCA497R | 1:100 |
|  | rabbit anti-CD163 | Santa Cruz Biotechnology, Santa Cruz, CA | Sc-33560 | 1:80 |
|  | rabbit anti-CD204 | GeneTex Inc., Hsinchu City, Taiwan | GTX51749 | 1:100 |
|  | HRP-anti-mouse/rabbit mix | DAKO, Glostrup, Denmark | K5007 |  |
| Flow cytometry | mouse anti-CD68 | BD Biosciences, San Jose, CA | 556059 | 1:50 |
|  | mouse anti-CD163 | BD Biosciences, San Jose, CA | 556017 | 1:50 |
|  | rabbit anti-CD204 | GeneTex Inc., Hsinchu City, Taiwan | GTX51749 | 1:200 |
|  | anti-mouse IgG-FITC | Santa Cruz Biotechnology, Santa Cruz, CA | sc-2099 | 1:100 |
|  | anti-rabbit IgG-PE | Santa Cruz Biotechnology, Santa Cruz, CA | sc-3745 | 1:100 |
| Immunoblot analysis | rabbit anti-HSP90α | GeneTex Inc., Hsinchu City, Taiwan | GTX109753 | 1:1000 |
|  | rabbit anti-JAK2 | Cell Signaling, Danvers, MA | 3230 | 1:1000 |
|  | rabbit anti-p-JAK2 | Cell Signaling, Danvers, MA | 3771 | 1:1000 |
|  | rabbit anti-TYK2 | Cell Signaling, Danvers, MA | 9321 | 1:1000 |
|  | rabbit anti-p-TYK2 | Cell Signaling, Danvers, MA | 9312 | 1:1000 |
|  | rabbit anti-STAT-3 | EMD Millipore, Billerica, MA | 04-1014 | 1:1000 |
|  | rabbit anti-p-STAT-3 | Epitomics, Burlingame, CA | 2236 | 1:500 |
|  | mouse anti-GAPDH | Novus Biologicals, Littleton, CO | NB300-221 | 1:20000 |
|  | HRP-anti-mouse | Santa Cruz Biotechnology, Santa Cruz, CA | sc-516102 | 1:5000 |
|  | HRP-anti-rabbit | Santa Cruz Biotechnology, Santa Cruz, CA | sc-2357 | 1:5000 |
| PLA | rabbit anti-HSP90α | AbD Serotec, Raleigh, NC | AHP-1339 | 1:80 |
|  | goat anti-TLR4 | Santa Cruz Biotechnology, Santa Cruz, CA | Sc-8694 | 1:40 |
|  | mouse anti-CD91 | BD Biosciences, San Jose, CA | 550495 | 1:80 |
|  | rabbit anti-MyD88 | Abcam, Cambridge, UK | ab2068 | 1:100 |
|  | goat anti-JAK2 | Santa Cruz Biotechnology, Santa Cruz, CA | sc-34480 | 1:40 |
|  | goat anti-TYK2 | Santa Cruz Biotechnology, Santa Cruz, CA | sc-30671 | 1:40 |
| ChIP | rabbit anti-STAT-3 | Cell Signaling, Danvers, MA | 12640 | 1:80 |
| Mouse therapy | mouse anti-HSP90α | LTK Biotechnologies, Taoyuan, Taiwan |  |  |

**Table S2.** The primers and PCR conditions adopted in this study. All reactions started at 95°C for 5 min and terminated at 72°C for 7 min.

| **Gene** | **Primer Sequence** | **PCR Condition & Product Size** |
| --- | --- | --- |
| EPB41L4A-AS1 | Forward: 5’-CAT-CGA-CTA-TGC-CAG-GGA-GT-3’  Reverse: 5’-TCC-ATC-TTC-CAC-CTG-TAG-GG-3’ | 95°C (30 sec), 58°C (40 sec), and 72°C (40 sec) for 31 cycles; 198 bp |
| ZNFX1-AS1 | Forward: 5’-CGG-CCT-GGA-CAA-CTA-CTA-GA-3’  Reverse: 5’-GAC-CCG-AAA-CGC-TCT-CTT-TC-3’ | 95°C (30 sec), 59°C (40 sec), and 72°C (40 sec) for 37 cycles; 101 bp |
| AC140481.1 | Forward: 5’-TCT-CTC-CTG-GAA-TGG-CAT-CT-3’  Reverse: 5’-GAG-GAA-CCC-CAT-GTA-AGC-AA-3’ | 95°C (30 sec), 58°C (40 sec), and 72°C (40 sec) for 34 cycles; 201 bp |
| NRSN2-AS1 | Forward: 5’-GAC-CAG-CAT-CTC-AAC-TGC-AA-3’  Reverse: 5’-TCT-TCC-CCT-CCT-CTT-CCA-AT-3’ | 95°C (30 sec), 58°C (40 sec), and 72°C (40 sec) for 38 cycles; 207 bp |
| RP11-608021 | Forward: 5’-TCC-TGA-GCA-AAC-AAC-AGA-CG-3’  Reverse: 5’-AAG-AAT-CAT-CAC-CGG-AGT-CG-3’ | 95°C (30 sec), 57°C (40 sec), and 72°C (40 sec) for 37 cycles; 163 bp |
| RP11-539E17.5 | Forward: 5’-TGG-GAT-GGA-CTG-CCT-AGA-AC-3’  Reverse: 5’-CCT-GAT-GTC-CCC-AGA-GAT-GT-3’ | 95°C (30 sec), 59°C (40 sec), and 72°C (40 sec) for 38 cycles; 226 bp |
| RP11-278L15.2 | Forward: 5’-AGT-TGG-GCC-ACC-TAC-ATC-AG-3’  Reverse: 5’-CTG-GCA-TGT-GAG-ATG-TGG-AG-3’ | 95°C (30 sec), 58.5°C (40 sec), and 72°C (40 sec) for 35 cycles; 207 bp |
| LINC00944 | Forward: 5’-CGA-ACC-CTT-CGA-TTG-AGA-AG-3’  Reverse: 5’-GCT-CGT-TCT-TCC-AGC-ATT-TC-3’ | 95°C (30 sec), 57°C (40 sec), and 72°C (40 sec) for 38 cycles; 192 bp |
| LINC00884 | Forward: 5’-TTC-CCT-CCG-CAG-AAG-ACT-TA-3’  Reverse: 5’-AAG-GCG-GCT-GAG-TTC-TGT-AA-3’ | 95°C (30 sec), 59°C (40 sec), and 72°C (40 sec) for 43 cycles; 229 bp |
| LINC00668 | Forward: 5’-TCC-CTG-CAA-CCT-TGA-ACT-CT-3’  Reverse: 5’-ATC-TGA-TCT-GCT-CCG-CAA-CT-3’ | 95°C (30 sec), 59°C (40 sec), and 72°C (40 sec) for 31 cycles; 147 bp |
| LINC00460 | Forward: 5’-CAT-GCA-CAC-TTC-TCG-GCT-AA-3’  Reverse: 5’-TTC-CCA-CGC-TCA-GTC-TTT-CT-3’ | 95°C (30 sec), 58°C (40 sec), and 72°C (40 sec) for 38 cycles; 176 bp |
| AC098973.2 | Forward: 5’-AAT-ATT-CGC-GTG-GGA-GTG-AC-3’  Reverse: 5’-TCT-TGC-CAG-GAG-TCA-GTC-AA-3’ | 95°C (30 sec), 59°C (40 sec), and 72°C (40 sec) for 41 cycles; 102 bp |
| HOTAIR | Forward: 5’-GCC-TTT-CCC-TGC-TAC-TTG-TG-3’  Reverse: 5’-AGA-GCT-TCC-AAA-GGC-TAG-GG-3’ | 95°C (30 sec), 59°C (40 sec), and 72°C (40 sec) for 38 cycles; 235 bp |
| CTD-3010D24.3 | Forward: 5’-GGG-TGC-ACA-GGG-TTA-TCA-GT-3’  Reverse: 5’-AGG-CAG-AGA-GGT-CAG-TTC-CA-3’ | 95°C (30 sec), 61°C (40 sec), and 72°C (40 sec) for 32 cycles; 178 bp |
| RP11-556E13.1 | Forward: 5’-ACT-GGA-TTT-GCA-GTG-CTG-TG-3’  Reverse: 5’-TCA-TGA-CGA-CAA-AGC-ACC-AT-3’ | 95°C (30 sec), 57°C (40 sec), and 72°C (40 sec) for 36 cycles; 108 bp |
| LOC100190940 | Forward: 5’-ATT-TCC-ATG-GCG-TTT-CTC-AC-3’  Reverse: 5’-AGG-CAG-GAG-AAT-TGC-TTG-AA-3’ | 95°C (30 sec), 57°C (40 sec), and 72°C (40 sec) for 39 cycles; 191 bp |
| LOC101927967 | Forward: 5’-CCA-GGC-TGG-AGT-GTA-ATG-GT-3’  Reverse: 5’-CTG-TAT-TCC-CGG-CTA-CTC-CA-3’ | 95°C (30 sec), 59°C (40 sec), and 72°C (40 sec) for 43 cycles; 102 bp |
| LOC283028 | Forward: 5’-CAC-CTG-CAC-ATT-TAG-GCT-CA-3’  Reverse: 5’-GGT-GGC-CAC-AGC-ATT-AAA-GT-3’ | 95°C (30 sec), 59.5°C (40 sec), and 72°C (40 sec) for 42 cycles; 284 bp |
| MNX1-AS1 | Forward: 5’-GCA-GCA-GAA-CTA-CCC-TCC-AG-3’  Reverse: 5’-ACC-CTC-TCC-TCC-TCC-CAC-TA-3’ | 95°C (30 sec), 62°C (40 sec), and 72°C (40 sec) for 37 cycles; 271 bp |
| LOC340340 | Forward: 5’-TGT-CAA-CAC-GAA-GGC-AGA-AG-3’  Reverse: 5’-GGG-ATA-TGC-CAA-CCT-TGA-GA-3’ | 95°C (30 sec), 57°C (40 sec), and 72°C (40 sec) for 38 cycles; 199 bp |
| CCAT1 | Forward: 5’-TTA-CTG-CCT-GAG-CTC-CAC-CT-3’  Reverse: 5’-ACG-GTG-ACA-GGT-CAT-TAG-GC-3’ | 95°C (30 sec), 60°C (40 sec), and 72°C (40 sec) for 36 cycles; 144 bp |
| CDKN2B-AS1 | Forward: 5’-AGG-AAA-GCG-AGG-TCA-TCT-CA-3’  Reverse: 5’-ATC-TGG-TGG-CCA-GAA-AAC-AG-3’ | 95°C (30 sec), 57°C (40 sec), and 72°C (40 sec) for 35 cycles; 288 bp |
| MGC16025 | Forward: 5’-CAG-TGA-GTC-CAG-GCT-CCT-TC-3’  Reverse: 5’-GTC-ACC-CTA-GAG-CTG-CCA-AG-3’ | 95°C (30 sec), 61°C (40 sec), and 72°C (40 sec) for 41 cycles; 198 bp |
| LOC101927256 | Forward: 5’-AGC-TGA-CTC-CCC-TCA-GAA-CA-3’  Reverse: 5’-TTC-CCT-CCC-ACA-CTG-GAT-AG-3’ | 95°C (30 sec), 59°C (40 sec), and 72°C (40 sec) for 37 cycles; 223 bp |
| GNG12-AS1 | Forward: 5’-CCA-GTG-CTT-CAA-AAT-CAG-CA-3’  Reverse: 5’-ATG-GGG-GCT-CTG-AGA-GAT-TT-3’ | 95°C (30 sec), 59°C (40 sec), and 72°C (40 sec) for 39 cycles; 231 bp |
| LOC441081 | Forward: 5’-ACG-GGC-TTT-CAC-TAT-GTT-GG-3’  Reverse: 5’-CCC-GTG-GAT-CAC-TGA-AGA-TT-3’ | 95°C (30 sec), 57°C (40 sec), and 72°C (40 sec) for 35 cycles; 154 bp |
| LINC00520 | Forward: 5’-ATG-AGA-TGT-CAG-CCC-CTC-AC-3’  Reverse: 5’-GTC-CAA-GGC-ACA-AGA-AGC-TC-3’ | 95°C (30 sec), 60°C (40 sec), and 72°C (40 sec) for 40 cycles; 198 bp |
| LOC101928152 | Forward: 5’-TGG-GAG-ATG-AAA-CAG-GAA-GC-3’  Reverse: 5’-ACC-AGC-CCA-TGA-CCA-AAA-TA-3’ | 95°C (30 sec), 57°C (40 sec), and 72°C (40 sec) for 28 cycles; 244 bp |
| ST7-AS2 | Forward: 5’-TCA-CCT-GTT-GGG-CAA-TAT-CA-3’  Reverse: 5’-TCC-CAT-TCC-AGT-GTG-TTT-CA-3’ | 95°C (30 sec), 56.5°C (40 sec), and 72°C (40 sec) for 40 cycles; 205 bp |
| GAPDH | Forward: 5’-GAA-GGT-GAA-GGT-CGG-AGT-3’  Reverse: 5’-GAA-GAT-GGT-GAT-GGG-ATT-TC-3’ | 95°C (30 sec), 56°C (40 sec), and 72°C (40 sec) for 25 cycles; 220 bp |
| α-SMA | Forward: 5’-TCC-AGA-GGC-ATA-GAG-AGA-CA-3’  Reverse: 5’-ACC-CTG-AAG-TAC-CCG-ATA-GA-3’ | 95°C (30 sec), 52°C (40 sec), and 72°C (40 sec) for 30 cycles; 222 bp |
| VE-cadherin | Forward: 5’-GTT-TCG-TGG-TGT-TAT-GTC-CT-3’  Reverse: 5’-AGT-TGT-TCC-GAG-TCA-CAA-AA-3’ | 95°C (30 sec), 49°C (40 sec), and 72°C (40 sec) for 32 cycles; 243 bp |
| CD31 | Forward: 5’-TGT-CTC-CAG-ACA-CCA-TTC-CA-3’  Reverse: 5’-TTA-GAG-CGC-CCT-CTT-GTG-TT-3’ | 95°C (30 sec), 52°C (45 sec), and 72°C (45 sec) for 25 cycles; 202 bp |
| Tie1 | Forward: 5’-AGT-TTC-GAG-GCT-GCT-CCA-3’  Reverse: 5’-TGT-GCT-GGT-CGG-AGA-GAA-3’ | 95°C (30 sec), 53°C (40 sec), and 72°C (40 sec) for 25 cycles; 282 bp |
| Tie2 | Forward: 5’-GGC-AAG-AAG-GAA-CAG-CAG-A-3’  Reverse: 5’-GCA-AAA-GCA-GCA-GCA-GAA-G-3’ | 95°C (30 sec), 53°C (40 sec), and 72°C (40 sec) for 25 cycles; 267 bp |
| Fibronectin | Forward: 5’-ACC-AAC-CTA-CGG-ATG-ACT-CG-3’  Reverse: 5’-GCT-CAT-CAT-CTG-GCC-ATT-TT-3’ | 95°C (30 sec), 56°C (40 sec), and 72°C (40 sec) for 33 cycles; 229 bp |
| IL-1β | Forward: 5’-ATT-CTG-ATG-AGC-AAC-CGC-TT-3’  Reverse: 5’-GCA-CAC-CAG-TCC-AAA-TTG-AA-3’ | 95°C (30 sec), 52°C (40 sec), and 72°C (40 sec) for 30 cycles; 156 bp |
| TNF-α | Forward: 5’-CCC-AGG-CAG-TCA-GAT-CAT-CTT-3’  Reverse: 5’-TCT-CAG-CTC-CAC-GCC-ATT-3’ | 95°C (30 sec), 62°C (40 sec), and 72°C (40 sec) for 26 cycles; 140 bp |
| CD163 | Forward: 5’-GAA-TAT-CAA-AAT-TGC-AAT-CAT-AGG-G-3’  Reverse: 5’-GTT-CAT-TTG-CTT-TGC-TTT-AGT-AAG-C-3’ | 95°C (30 sec), 62°C (40 sec), and 72°C (40 sec) for 32 cycles; 265 bp |
| CD204 | Forward: 5’-ATA-GCT-TCC-AGA-TTA-CAA-AGG-CC-3’  Reverse: 5’-AGA-AAA-ATA-AGC-TCC-CTG-GTC-C-3’ | 95°C (30 sec), 56°C (40 sec), and 72°C (40 sec) for 32 cycles; 328 bp |
| IL-10 | Forward: 5’-ATG-CCC-CAA-GCT-GAG-AAC-CAA-GAC-CCA-3’  Reverse: 5’-TCT-CAA-GGG-GCT-GGG-TCA-GCT-ATC-CCA-3’ | 95°C (30 sec), 60°C (40 sec), and 72°C (40 sec) for 30 cycles; 365 bp |
| TGF-β | Forward: 5’-CTA-CTA-CGC-CAA-GGA-GGT-CAC-3’  Reverse: 5’-TTG-CTG-AGG-TAT-CGC-CAG-GAA-3’ | 95°C (30 sec), 60°C (40 sec), and 72°C (40 sec) for 30 cycles; 249 bp |
| Arg1 | Forward: 5’-ATG-CAG-AAC-TGT-GTG-GCA-TG-3’  Reverse: 5’-GGG-TTG-GTT-GAT-AAA-AGG-CA-3’ | 95°C (30 sec), 56°C (40 sec), and 72°C (40 sec) for 36 cycles; 254 bp |
| iNOS | Forward: 5’-GAG-AAA-GCC-CCC-TGT-GCC-3’  Reverse: 5’-TAC-CGC-TTC-CAC-CCT-GGC-3’ | 95°C (30 sec), 57°C (40 sec), and 72°C (40 sec) for 35 cycles; 422 bp |
| HSP90α | Forward: 5′-GCA-GAG-AAA-ACT-CTG-TCT-CG-3′  Reverse: 5′-CCT-GAG-AGA-ATT-CAC-TGT-GAG-C-3′ | 95°C (30 sec), 55°C (40 sec), and 72°C (40 sec) for 28 cycles; 163 bp |
| IL-13 | Forward: 5’-GTA-CTG-TGC-AGC-CCT-GGA-AT-3’  Reverse: 5’-TTT-ACA-AAC-TGG-GCC-ACC-TC-3’ | 95°C (30 sec), 60°C (40 sec), and 72°C (40 sec) for 30 cycles; 162 bp |
| IL-4 | Forward: 5’-GCC-ACC-ATG-AGA-AGG-ACA-CT-3’  Reverse: 5’-ACT-CTG-GTT-GGC-TTC-CTT-CA-3’ | 95°C (30 sec), 60°C (40 sec), and 72°C (40 sec) for 32 cycles; 152 bp |
| CCL22 | Forward: 5’-AGT-AAA-TAC-TTA-AGA-GGC-CAA-3’  Reverse: 5’-GCA-GAC-ACT-ATT-TTA-AAG-GAT-3’ | 95°C (40 sec), 51°C (40 sec), and 72°C (40 sec) for 30 cycles; 828 bp |
