# Supplementary material for: Endothelial-mesenchymal transition harnesses HSP90α-secreting M2-macrophages to exacerbate pancreatic ductal adenocarcinoma
Source: J Hematol Oncol. 2019 Dec 17;12:138. doi: 10.1186/s13045-019-0826-2 (PMC6918594; doi:10.1186/s13045-019-0826-2)
Supplement: Supplementary file 2 — Additional file 2: Figure S1. OPN induces EndoMT. A, mRNA levels of VE-cadherin, CD31, and α-SMA in the HUVECs pre-incubated 16 h with 2% FBS-containing M199 medium and then added with PBS or 0.3 μg/ml of OPN for further 24 h. We have previously reported that OPN induced EndoMT of HUVECs and immortalized endothelial cell line EC-RF24 (12). Consistently, the present data shows that OPN induced down-regulation of endothelial cell marker genes (VE-cadherin and CD31) but up-regulation of mesenchymal cell marker gene α-SMA. B, mRNA levels of VE-cadherin, Tie1, Tie2, CD31, α-SMA, and fibronectin in mouse immortalized endothelial cell line 3B-11 pre-incubated 16 h with 1% FBS-containing RPMI 1640 medium and then added with PBS or 0.3 μg/ml of OPN for further 24 h. The data revealed that OPN also induced EndoMT of 3B-11 cells. Figure S2. Using TGF-β-induced EndoMT model to confirm EndoMT CM-induced marophage M2-polarization. EndoMT CM and control medium (CTRL) were prepared as described in the Methods section except 20 ng/ml of TGF-β was used instead of OPN. THP-1-derived macrophages were treated with CTRL or EndoMT CM for 24 h. Relative mRNA levels of IL-1β, TNF-α, iNOS, CD163, CD204, IL-10, TGF-β, and Arg1 were assessed by qPCR analyses. #, P < 0.001 when compared with CTRL. Figure S3. eHSP90α enhances the physical association of CD91 with TLR4. PLAs showed red fluorescent dots in PBS or rHSP90α-treated macrophages by using the antibody combination detecting the physical interaction of CD91–TLR4. The level of red fluorescent dots was increased upon rHSP90α treatment. [file 13045_2019_826_MOESM2_ESM.docx]

**Additional File 2**

| **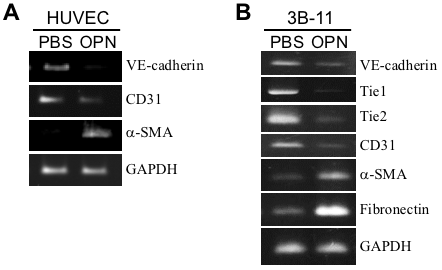** |
| --- |
| **Figure S1.** OPN induces EndoMT. **A,** mRNA levels of VE-cadherin, CD31, and α-SMA in the HUVECs pre-incubated 16 h with 2% FBS-containing M199 medium and then added with PBS or 0.3 μg/ml of OPN for further 24 h. We have previously reported that OPN induced EndoMT of HUVECs and immortalized endothelial cell line EC-RF24 (12). Consistently, the present data shows that OPN induced down-regulation of endothelial cell marker genes (VE-cadherin and CD31) but up-regulation of mesenchymal cell marker gene α-SMA. **B,** mRNA levels of VE-cadherin, Tie1, Tie2, CD31, α-SMA, and fibronectin in mouse immortalized endothelial cell line 3B-11 pre-incubated 16 h with 1% FBS-containing RPMI 1640 medium and then added with PBS or 0.3 μg/ml of OPN for further 24 h. The data revealed that OPN also induced EndoMT of 3B-11 cells. |

| **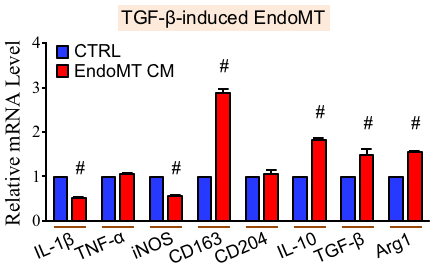** |
| --- |
| **Figure S2.** Using TGF-β-induced EndoMT model to confirm EndoMT CM-induced marophage M2-polarization. EndoMT CM and control medium (CTRL) were prepared as described in the Methods section except 20 ng/ml of TGF-β was used instead of OPN. THP-1-derived macrophages were treated with CTRL or EndoMT CM for 24 h. Relative mRNA levels of IL-1β, TNF-α, iNOS, CD163, CD204, IL-10, TGF-β, and Arg1 were assessed by qPCR analyses. #, *P* < 0.001 when compared with CTRL. |

| **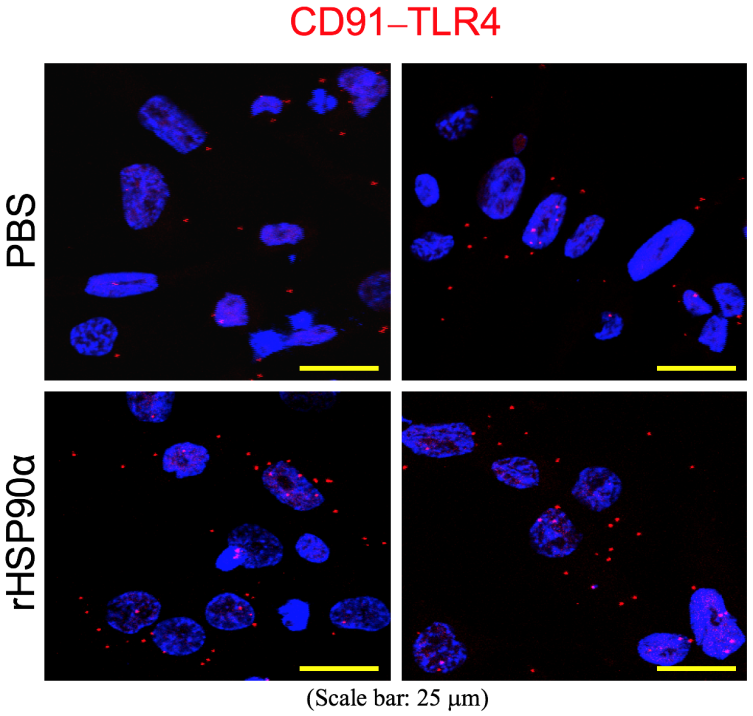** |
| --- |
| **Figure S3.** eHSP90α enhances the physical association of CD91 with TLR4. PLAs showed red fluorescent dots in PBS or rHSP90α-treated macrophages by using the antibody combination detecting the physical interaction of CD91–TLR4. The level of red fluorescent dots was increased upon rHSP90α treatment. |
